# A Critical Evaluation of the Limiting Current Density in Polymer Electrolytes: Interplay of Ion Transport, Mechanical Stability, and Conformal Li–Electrolyte Interfaces

**DOI:** 10.1021/jacs.5c16267

**Published:** 2026-01-23

**Authors:** Philipp Röring, Jan Pleie, Andreas J. Butzelaar, Gerrit M. Overhoff, Christina Schmidt, Kerstin Neuhaus, Patrick Théato, Martin Winter, Gunther Brunklaus

**Affiliations:** † 28334 Forschungszentrum Jülich GmbH, Helmholtz-Institute Münster (IMD-4), Corrensstraße 46, 48149 Münster, Germany; ‡ University of Münster, Institute of Physical Chemistry/MEET Battery Research Center, Corrensstraße 46, 48149 Münster, Germany; § University of Münster, International Graduate School for Battery Chemistry, Characterization, Analysis, Recycling and Application (BACCARA), Corrensstr. 40, 48149 Münster, Germany; ∥ Karlsruhe Institute of Technology (KIT), Institute for Chemical Technology and Polymer Chemistry (ITCP), Engesserstraße 18, 76131 Karlsruhe, Germany; ⊥ Karlsruhe Institute of Technology (KIT), Soft Matter Laboratory-Institute for Biological Interfaces III (IBG-3), Hermann-von-Helmholtz-Platz 1, 76344 Eggenstein-Leopoldshafen, Germany

## Abstract

Solid-state batteries with lithium metal anodes are among the promising candidates to fulfill the actual requirements of growing energy demands in comparison to commercially available lithium-ion batteries, despite the current challenges of inhomogeneous lithium metal deposition upon cycling. In the present literature, the limiting current density is referred to as a key performance indicator for faster charging of solid-state batteries, though from a practical point of view, it is defined as the maximum endurable current density that might be applied without possible cell failure. In this study, we evaluate the obtained values of limiting current densities for lithium metal batteries operating with polymer electrolytes. Notably, we critically compare various experimental procedures to determine the actual limiting current density and discuss the impact of external factors such as scan rate, temperature, and applied cell pressure, thereby invoking model-type PEO-based electrolytes to examine available mechanical properties that may afford suppression of lithium dendrite formation. In fact, we demonstrate that experimentally derived limiting current densities are not intrinsic electrolyte characteristics but rather entities strongly dependent on the applied conditions and hence should ideally be determined based on different techniques to deliver meaningful data.

## Graphical abstract

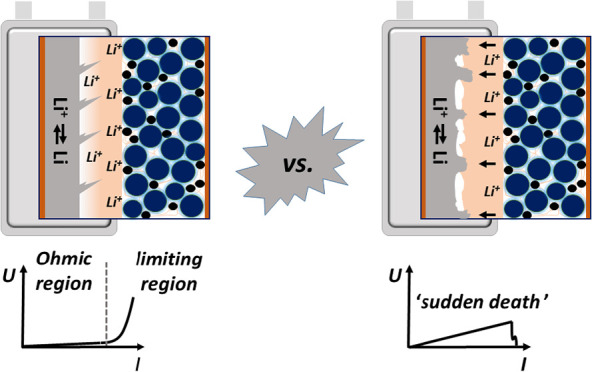

## Introduction

Solid-state lithium metal batteries with ultrathin lithium metal anodes are of particular interest since they theoretically offer boosted energy density and faster charge capability than commercially available lithium-ion batteries.^1,2^ A key to potentially enable solid-state batteries comprise tailored solid electrolytes, which can be divided into ceramic and polymer electrolytes.^3,4^ In general, solid polymer electrolytes (SPE) are compatible with a variety of electrode materials, provide good wettability, and often can be readily processed.^5^ Currently, many candidate polymer electrolytes suffer from unfavorable ionic conductivity and insufficient contacts with thick porous electrodes.^6^ In addition, a yet remaining technical challenge for lithium-metal-based solid-state batteries includes inhomogeneous lithium deposition, particularly at higher current densities and in the presence of nonideal “roughened” electrode-**|**electrolyte interfaces.^7–9^ Here, lithium-ion transport within solid-state electrolytes as well as charge transfer across interfaces (as determined by lithium diffusivity) denote crucial aspects that affect the occurrence of lithium metal “dendrites”.^10,11^ Note that Bai et al. describe lithium metal deposition and potential onset of electrolyte mass transport limitations invoking Sand’s model, allowing for visualization of changes of lithium metal deposition from root-growing “mossy” lithium to tip-growing “dendritic” lithium at cell operating conditions where charge carrier depletion occurs at electrode interfaces.^12^ More recently, practical relevance of Sand’s equation for assessing polymer-based electrolytes was demonstrated.^13,14^

Inhomogeneous lithium metal deposition due to concentration polarization may result in cell failure, even in cases where the applied current density is below a limiting current density (LCD).^10^ Contact losses between solid-state electrolyte and lithium metal anode might occur,^15,16^ resulting in undesired void formation and uneven current distributions that promote inhomogeneous lithium metal deposition.^17^ The latter eventually yields “dendritic” lithium protrusions that may penetrate mechanically insufficiently robust polymer electrolytes, in this way short-circuiting the cells.^17,18^ Based on model predictions, Monroe and Newman suggested that a growth of lithium protrusions in the case of polymer electrolytes might be prevented provided that respective polymer membranes could afford shear moduli of *G* ^SPE^ > 2 *G* ^Li^ (*G* ≈ 6 GPa).^19–21^ In practice, reported data by Khurana et al. revealed sufficient dendrite growth resistance for PEO-based electrolytes already at moderate shear moduli in the order of ∼0.1 MPa, in this way bestowing longer lifetimes of lithium metal batteries.^22^ These observations manifest the understanding that lithium metal deposition may not only be governed by mechanical features and intrinsic properties of the polymer electrolytes but also could be affected by external operating conditions such as the applied temperature or pressure. Technically relevant in view of establishing fast charge condition upon cell operation, the limiting current density is defined as the maximum applicable current density that is tolerated by the cells without causing failure, e.g., due to any occurrence of lithium metal “dendrites” or protrusions.^10^ Various experimental procedures are available to derive the LCD, including dynamic experiments such as linear sweep voltammetry (LSV)^23–26^ or current scans (CS).^27,28^ Here, either the voltage or the applied currents are swept with a set sweep rate from open circuit to predefined cutoff values. Ideally, the current/voltage response follows Ohmic behavior at lower voltage/current excitations (Figure 1a,b), while at a threshold, the curve reflects a steady state, indicating ion depletion at lithium metal surfaces. This plateau (for LSV, see Figure 1a) or point of steep increase (under CS, cf. Figure 1b) is interpreted as the LCD. An alternative approach to estimate current limitations comprises galvanostatic experiments in which a constant current density is applied for a certain time or capacity. After lithium plating, similar amounts of lithium metal (either time- or capacity-controlled) are stripped again,^10,29^ and typically this cycle is repeated several times prior to increasing the applied current density, e.g., in steps of 0.1 mA cm^–2^. In general, voltage profiles reveal an IR drop at the beginning of each lithium plating or stripping step, attributed to the Ohmic cell resistance, reaching a steady-state plateau after some time (Figure 1c). If the charge carrier transport within the electrolytes is slower than continued lithium metal deposition from a reduction of lithium ions at the electrode-**|**electrolyte interfaces, then the voltage further increases. This so-called concentration polarization is a consequence of charge carrier depletion occuring in the vicinity of the electrode, which ultimately yields cell failure, eventually even in the case of single-ion conducting polymer electrolytes (Figure 1c).^30^

**Figure 1:**
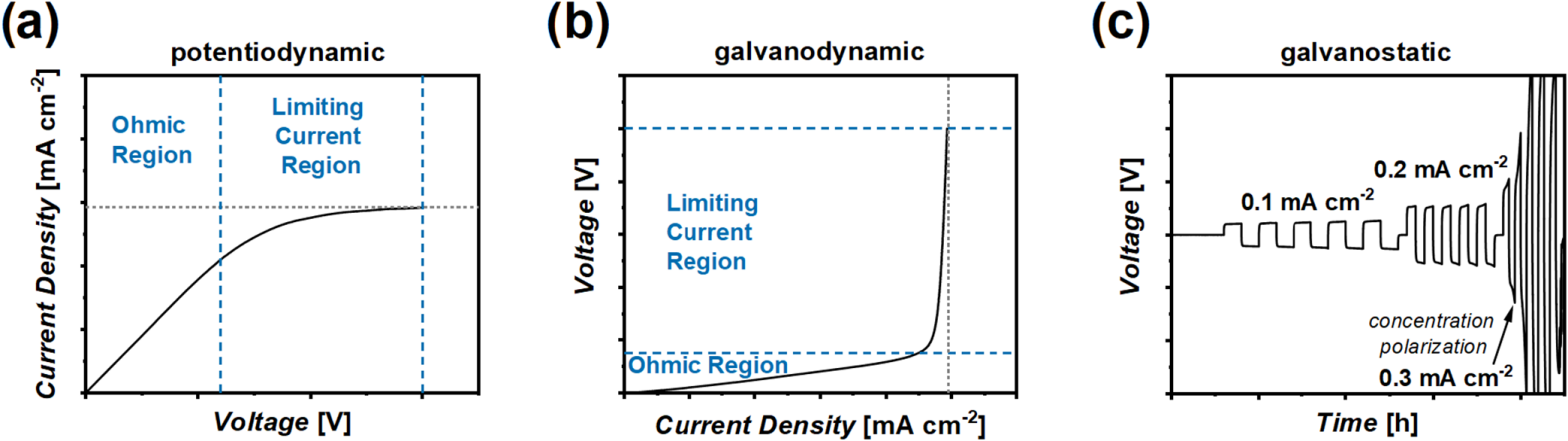
Experimental procedures to ideally determine a limiting current density: (a) Linear sweep voltammetry, (b) current scans, and (c) galvanostatic lithium metal plating/stripping experiments at increasing current densities.

In the reported literature, solely one of the introduced approaches is typically utilized to obtain the respective limiting current density of solid-state electrolytes. Nevertheless, to the best of our knowledge, there is no commonly accepted standardized guideline on how to derive the LCD, so experimentalists often invoke individual test protocols, rendering meaningful comparison of provided data for cells operated with various electrolytes challenging, particularly considering that salient parameters might have been unintentionally neglected.

Hence, in the present study, we systematically explore the impact of often ignored test parameters on the experimental values of LCDs, including external factors such as temperature and cell stack pressures, in addition to mechanical properties of solid polymer electrolytes. Besides, theoretical predictions are discussed in view of the derived values, thereby emphasizing the importance of the utilized conditions and interplay of these characteristics for the evaluation of polymer electrolytes for their suitability for fast charge applications. Notably, sweep-rate-dependent transport limitations are not unique to polymer solid-state electrolytes but represent a general feature of electrochemical systems, including lithium-metal cells with liquid electrolytes, where diffusion-limited currents governed by Sand-type behavior may be transiently exceeded.^12^

## Experimental Section

### Materials

Poly­(ethylene oxide) (PEO, *M*_n_ = 5,000,000 g mol^–1^, Aldrich) and benzophenone (BP, Aldrich) were both dried at 40 °C under reduced pressure (1 × 10^–3^ mbar) for a duration of 5 days. Bis­(trifluoromethane)­sulfonimide lithium salt (LiTFSI, purity = 99.95%, Aldrich) was dried at 120 °C under reduced pressure (1 × 10^–3^ mbar) for 2 days and tantalum-doped lithium lanthanum zirconate (LLZO, Li_6.4_La_3_Zr_1.4_Ta_0.6_O_12_, purity = >99.90%, particle size (D50) = 400–600 nm, Ampcera) was dried at 130 °C under reduced pressure (1 × 10^–8^ mbar) for 3 days. All chemicals should be moisture-free, as defined by residual moisture concentrations below 20 mg kg^–1^ (ppm). Drying under reduced pressure and with increased temperature effectively reduces the residual moisture content.^31–33^ PEO, BP, LiTFSI, and LLZO were subsequently stored in a glovebox (MBraun Unilab, <0.1 ppm of H_2_O, <0.1 ppm of O_2_) under an inert argon atmosphere. The block copolymer poly­(vinyl benzyl methoxy poly­(ethylene oxide) ether-*block*-polystyrene) was prepared according to previous work^23^ and stored inside the glovebox (MBraun Unilab, <0.1 ppm of H_2_O, <0.1 ppm of O_2_). Acetonitrile (anhydrous, 99.90% purity, Aldrich) was used as received; lithium metal (300 μm, Honjo lithium) was stored in a glovebox (MBraun Unilab, <0.1 ppm of H_2_O, <0.1 ppm of O_2_) and utilized without further surface modification.

### Solid Polymer Electrolyte (SPE) Preparation

#### Preparation of SPE Based on Linear PEO

For the preparation of the SPE based on linear PEO, a [Li^+^]/[EO] ratio of 1:10 was achieved for all of the different model-type SPEs, so that 0.605 g of PEO and 0.395 g of LiTFSI were weighed. For cross-linked PEO, 0.05 g of BP (8.25 wt % referring to the polymer weight) and for PEO-LLZO, 0.05 g of LLZO (5 wt % referring to the total weight) were added. All ingredients were mortared until a homogeneous, cotton-like powder could be achieved. The powder was formed to a “ball” and vacuum-sealed within a pouch-foil, which was then placed and heated in an oven at 100 °C for 2 days. After removing the mixture from the pouch-foil, it was hot-pressed (100 °C, 10 bar, 5 min, followed by 100 °C, 100 bar, 5 min) to a flat membrane (100 μm thickness). The membrane containing BP was afterward placed under a UV lamp (Hönle UVACUBE 100) for a duration of 5 min to initialize cross-linking to yield a dense network. All work was conducted in a dry room (dew point = −65 °C, relative humidity = 0.022%) to avoid any contamination with residual moisture.

#### Preparation of SPE Based on the Block Copolymer

Prior to the SPE membrane preparation, the block copolymer was dried at 80 °C under reduced pressure (1 × 10^–3^ mbar) overnight. The polymer and the corresponding amount of LiTFSI with a predefined ratio of [Li^+^]/[EO] 1:10 were dissolved in acetonitrile (MeCN, 3 mL). Subsequently, the solution was poured into a Teflon mold. The mold was covered, and MeCN was allowed to slowly evaporate at ambient conditions enabling nanophase separation. Subsequently, the obtained SPE film was dried under annealing at 110 °C and reduced pressure (1 × 10^–3^ mbar) for 24 h. The film thickness of the resulting membrane was approximately 100 μm.

### Coin Cell Assembly

For all of the electrochemical measurements, Li||Li cells were assembled. A coin-cell-type (CR2032) two-electrode setup was applied with two lithium metal discs (Honjo lithium, thickness = 300 μm, Ø = 13 mm) separated by one of the PEO-based model-type solid polymer electrolytes (thickness = ∼100 μm, Ø = 14 mm). Different stainless-steel spacers were used to adjust and maintain the cell stack pressure in the coin cell, where the pressure was simply increased by keeping the stack thickness using thicker stainless-steel spacers. Note that more detailed information on the derivation of the explicit cell stack pressure are summarized in the Supporting Information.

### Electrochemical Measurements

#### Potentiodynamic Experiments

All of the potentiodynamic experiments were performed in a two-electrode setup using a VMP3 multichannel potentiostat of Biologic. When not explicitly mentioned, the temperature was held constant at 60 °C; all of the cells were conditioned at the respective temperature for 12 h prior to the measurements. The open circuit voltage (OCV) was recorded during a 12 h conditioning-step and no significant change was monitored (please also see Figure S3 in the Supporting Information). Note that to determine the limiting current density via linear sweep voltammetry (LSV), a voltage sweep was conducted at sweep rates of either 1.0 mV s^–1^ or 0.02 mV s^–1^ until a cutoff voltage of 1.0 V vs Li|Li^+^ was reached. A plateau or a sudden cell short circuit indicates the actual value of the LCD. For each of the SPEs and at different conditions, three cells were assembled, and the respective values of LCD were obtained as an average value, whereas only one representative cell is shown in the graph.

For the measurement of the LCDs via current scans (CS), a current ramp at a sweep rate of either 1.0 μA s^–1^ or 0.02 μA s^–1^ was applied until a current density of 5.0 mA cm^–2^ or a cutoff voltage of 5.0 V vs Li|Li^+^ was reached. Here, a steep voltage increase or a sudden voltage drop indicates the respective LCD. For all of the SPEs and different conditions, three cells were assembled, and the value of LCD was calculated as mean, whereas only one representative cell is shown in the graph.

For both, LSV and CS, impedance spectra were collected in a frequency range of 1 MHz to 100 mHz at an excitation amplitude of 10 mV before and after the 12 h conditioning-step as well as prior to and after a voltage or current ramp.

Cyclic voltammetry (CV) experiments were performed between an upper cutoff voltage of 1.0 V vs Li|Li^+^ and a lower cutoff voltage of −1.0 V vs Li|Li^+^. Different sweep rates were utilized, as indicated in the respective figures. The sweep rate was changed from high rates to low rates, and one cycle was conducted at each rate.

### Constant Current Cycling Experiments

All of the constant current cycling experiments were done in a coin-cell-type two-electrode setup using a Maccor series 4000 battery cell test system; the cells were conditioned at 60 °C in a climate chamber (Binder KB 400). For long-term metal deposition experiments, lithium was plated at a constant current density of 0.1 mA cm^–2^ until a sudden voltage drop reflects a cell shortening.

### Physicochemical Measurements

#### Oscillatory Rheology

Rheological data were recorded on a stress-controlled MCR 301 (Anton Paar) rheometer via oscillatory shear experiments. The sample membranes were prepared according to a previously introduced technique but at a thickness of 0.5 mm; round discs with Ø = 15 mm were punched out for the experiments. For determining the overall storage or loss modulus, a frequency sweep from 0.1 to 100 rad s^–1^ at a constant amplitude of 0.1% was used at 20 °C, 40 °C, and 60 °C. A constant force of 1 N was applied to ensure good interfacial contacts and reproducible pressure. The sample temperature was held constant for a duration of 5 min before applying a frequency sweep; all of the samples were analyzed under a nitrogen atmosphere.

### Simulation

The simulations are based on the theory of concentrated solutions developed by John S. Newman and others,^34,35^ utilizing the software COMSOL Multiphysics 6.0, considering model Li||Li cells, where the lithium metal electrodes are represented as two-dimensional surfaces that function as both an ion source and an ion sink. The model accounts for lithium-ion migration and diffusion within the polymer electrolyte, with charge-transfer kinetics at the electrodes described by the Butler–Volmer equation and relevant material parameters (conductivity, transference number, diffusion coefficient) taken from experimental measurements. Convection and mechanical effects of the electrolyte are not considered. Further details are provided in the Supporting Information.

## Results and Discussion

In this work, the impact of test parameters for the measurement of limiting current densities based on dynamic experiments is discussed. Both, linear sweep voltammetry and current scans were employed to obtain a comprehensive overview on how experimental conditions may alter the outcome. Two different sweep rates (1 μA s^–1^ and 0.02 μA s^–1^) were applied to examine changes of resulting LCDs by any ion transport limitations. Figure 2 displays the different dynamic measurements of Li||Li cells operated with cross-linked PEO at 60 °C. At fast sweep rates, a plateau or a steep increase could be observed for both methods, LSV and CS, at a current density of 0.92 ± 0.14 mA cm^–2^ and 0.96 ± 0.02 mA cm^–2^, likely resulting from ion depletion, hence indicating the values of the LCDs. At rather slow sweep rates, the anticipated Ohmic behavior is monitored at the beginning, though a sudden current peak or voltage drop was already observed at current densities of 0.41 ± 0.03 mA cm^–2^ or 0.45 ± 0.03 mA cm^–2^, respectively. The LCDs derived from the experiments at high sweep rates could not be reached at lower rates, and instead of a typical steady-state current density with drastically increased voltage, random voltage behavior was monitored.

**Figure 2:**
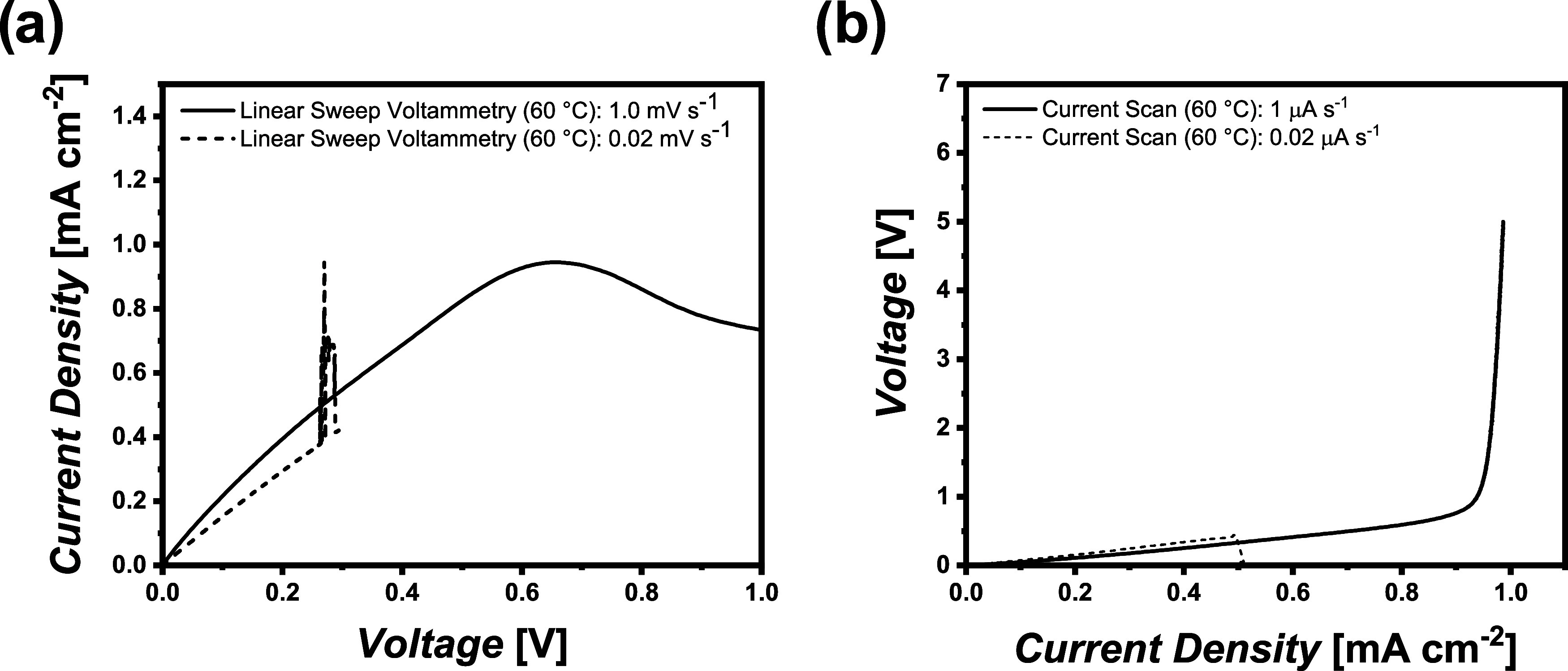
Different sweep rates in dynamic experiments of Li||Li cells with cross-linked PEO at 60 °C. (a) Linear sweep voltammetry and (b) current scans.

To further establish the impact of sweep rates on the experimentally determined limiting current densities, cyclic voltammetry (CV) with alternating sweep rates was applied to Li||Li cells, in this way emphasizing a correlation of peak current and sweep rate, as predicted by the Randles–Sevcik eq 1 ^36,37^

1 $$I_{p}=0.4463\cdot \sqrt{\frac{z^{3}F^{3}}{\mathrm{RT}}}\cdot A\cdot c_{0}\cdot \sqrt{D\nu }$$

where *I*_p_ denotes the peak current for oxidation or reduction, *z* reflects the number of electrons participating in the redox-active processes (Li|Li^+^), *F* and *R* represent the Faraday and universal gas constants, respectively, *T* represents the temperature, *A* is the electrode area (indeed assuming “ideal” contacts between electrode and electrolyte, that is, absence of excessive surface “roughness”), *c*_0_ is the molar concentration of LiTFSI present in the electrolyte prior to any concentration gradient that might be established, *D* is the diffusion coefficient, and **ν** denotes the sweep rate applied in the CV measurements. Note that eq 1 is strictly valid only if two conditions are met: (i) the redox reaction can be described as a quasi-reversible process and (ii) the diffusion is semi-infinite. Besides, most publications referring to the Randles–Sevcik model focus on comparatively slow solid-state diffusion of lithium-ions within the different cathode active materials.^38–41^ The CV plots and the relationship between the observed peak current and the square root of the applied scan rate are depicted in Figure 3. The linearity of the respective data point of the peak current vs the square root of the scan rate verifies the quasi-reversibility of the redox-active species and the condition of semi-infinite diffusion (Figure 3b). Considering that both conditions are fulfilled, we identified that the peak current increases while increasing the sweep rate. Basically, in a Li||Li cell, the peak current mirrors the limiting current density of the electrolyte. In comparison, the peak current at a scan rate of 1 mV s^–1^ (Figure 3a) amounts to 1.28 ± 0.03 mA (0.97 ± 0.02 mA cm^–2^), which is in good agreement with the experimentally determined LCD from previous experiments at the applied sweep rate (Figure 2). When decreasing the sweep rate, the peak current is also reduced. At a sweep rate of 0.25 mV s^–1^, the cells show minor voltage noises, and when further reducing the sweep rate to 0.1 mV s^–1^, a cell short circuit appears, in agreement with the sweep experiments at a sweep rate of 0.02 mV s^–1^ or 0.02 μA s^–1^. This corroborates that not only can charge carrier depletion at the vicinity of the (lithium) electrodes induce diffusion limits and dendrite growth but also cell features such as the mechanical stability of electrolytes might affect the derived values for the LCD. Note that the sweep rate governs the necessary measurement times. A rather slow sweep rate requires comparably long measurement times, resulting in increased amounts of charge passing through a system and hence yielding an increased amount of plated lithium.

**Figure 3:**
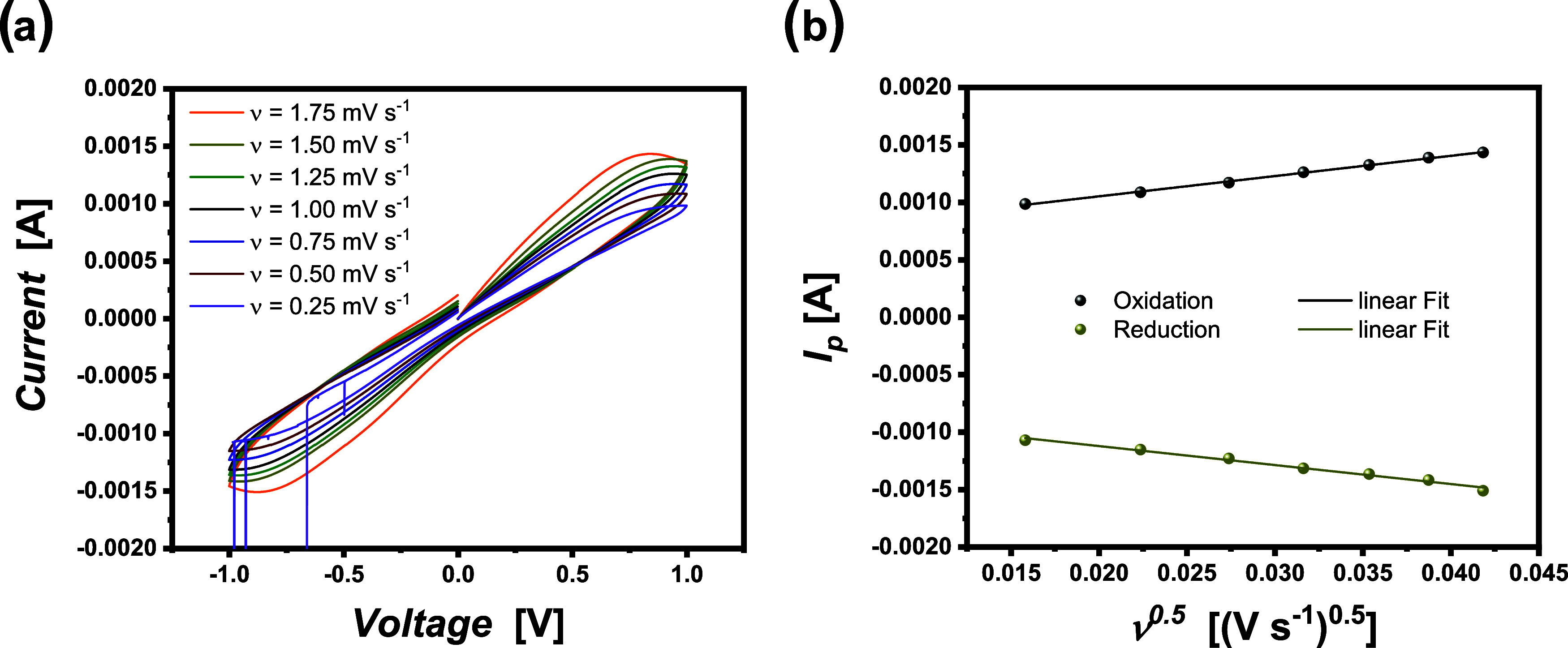
Dependence of peak currents from the sweep rate in cyclovoltammetry: (a) cyclic voltammogram for a Li||Li cell in the set voltage range from −1 to 1 V with alternating sweep rates. (b) Randles–Sevcik-type behavior of the peak current versus the square.

Below a critical sweep rate, current scan experiments approach a quasi-steady state and yield identical limiting current densities independent of the applied sweep rate. This critical sweep rate threshold may be approximated. If the applied current density *i*, when equal to the limiting current density *i*_lim_, changes slowly enough for charge transport to relax the present system to a current-dependent steady state, then the sweep rate ν is slow enough to reveal that same limiting current density. The condition is fulfilled if

2 $$\frac{\Delta i}{i_{\lim}}=\frac{\nu \cdot t}{i_{\lim}}\ll 1$$

where Δ*i* denotes a change in current density and t is the time that must pass for the system to relax to a steady state. One can therefore define a boundary sweep rate ν_lim_ below which all sweep rates ν that satisfy

3 $$\nu \ll \nu_{\lim}=\frac{i_{\lim}}{t}$$

reveal the same limiting current. The applied current density is approximately uniform over laterally homogeneous planar electrodes, and any residual lateral effects (e.g., any edge effects or contact inhomogeneities) are assumed to remain spatially localized and decay over length scales comparable to their lateral extent. Thus, the ion transport within the bulk electrolyte can be well approximated as one-dimensional normal to the electrodes, particularly when the lateral electrolyte extent (Ø = 14 mm) is much larger than the thickness of the electrolyte (*L* = 100 μm). If we equate the thickness of the electrolyte *L,* that is the length along which concentration must relax to reach a quasi-steady state, with the one-dimensional root mean squared displacement, as derived for a 1D random walk diffusion,^42^ then it provides a relation between the effective diffusion constant *D* and time *t*

4 $$L=\sqrt{2\mathrm{Dt}}\to t=\frac{L^{2}}{2D}$$

If we now combine this with eq 3 and an estimate for the limiting current density based on dilute solution theory^35^

5 $$i_{\lim}\approx \frac{4\mathrm{FD}c}{L}$$

where c reflects the salt concentration of the electrolyte, we obtain an estimate for the sweep rate threshold:

6 $$\nu_{\lim}\approx 8\cdot F\cdot D^{2}\cdot \frac{c}{L^{3}}$$

Invoking the experimentally fitted diffusion coefficient, ν_lim_ is calculated as 0.3 μA s^–1^ in the case of cross-linked PEO at 60 °C. Consequently, sweep rates ν ≪ ν_lim_ (e.g., 0.02 μA s^–1^, as employed in this work) are expected to yield an identical, transport-limited LCD value. In contrast, higher sweep rates (e.g., 1 μA s^–1^, also used in this work) increasingly probe higher current densities. To illustrate this behavior, a COMSOL simulation was carried out with different sweep rates, and the results are displayed in Figure 4. Sweep rates much smaller than the sweep rate threshold, e.g., 0.02 μA s^–1^ and smaller, all yield the same limiting current density.

**Figure 4:**
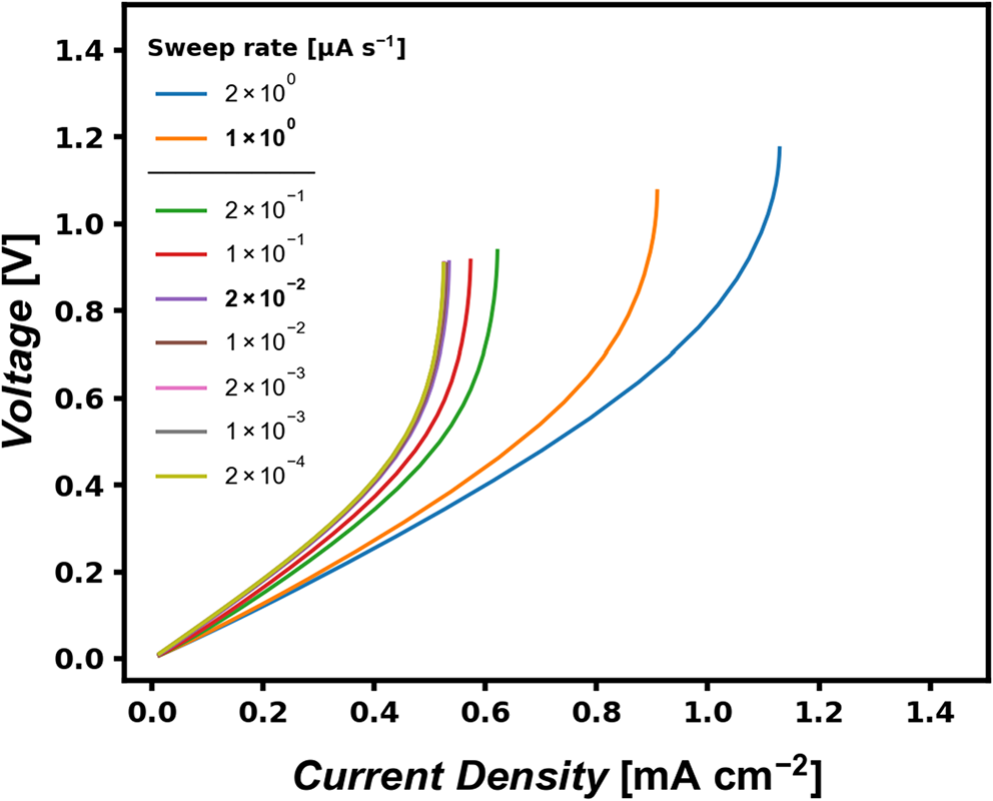
Simulated current sweep experiments showing the dependence of the sweep rate on the apparent limiting current density. For sweep rates much smaller than the upper bound, the extracted LCD converges to a sweep-rate-independent value, whereas higher sweep rates progressively overestimate the transport-limited current.

While this establishes transport-controlled sweep rate regimes and the convergence of LCDs at sufficiently slow sweep rates, experiments conducted at slow sweep rates (see, e.g., Figure 2b) exhibit premature voltage drops and cell short circuits at current densities well below those of the predicted LCDs. To elucidate the origin of this discrepancy, we next compare current scan experiments in Li||Li cells with COMSOL simulations considering only ion transport, without incorporating mechanical stability or any impact of nonuniform lithium deposition. Figure 5a displays the COMSOL simulated voltage profiles of current sweep experiments compared to experimental data at 60 °C. At a sweep rate of 1 μA s^–1^ (upper part of Figure 5a), the simulated and experimentally derived voltage curves are in good agreement, attributed to “ideal” experimental conditions. However, at a sweep rate of 0.02 μA s^–1^ (lower part of Figure 5a), the simulated data do not fully coincide with experimental data. While simulation reveals the typical steep increase in voltage at conditions where ion depletion likely occurs (as expected for such type of electrolytes), experimental data of Li||Li cells suffer from sudden voltage drop already at a current density of 0.45 ± 0.03 mA cm^–2^, which is visibly before the current density at which the occurrence of ion depletion is predicted. Note that the COMSOL simulations merely reflect ion transport characteristics such as ionic conductivity or transference numbers but disregard mechanical properties of the electrolyte itself or any nonuniformity of plated lithium metal. Therefore, such deviations between simulation and experiment are presumed not to be an effect of transport properties but rather due to mechanical issues of the polymer electrolytes. While electrochemical interphase formation and reactivity govern lithium deposition behavior and polymer degradation pathways may differ among the considered electrolytes, the present analysis focuses on ion transport limitations and mechanical stability, as these effects dominate the observed deviations between slow sweep rate experiments and simulation. It is not the scope of this work to investigate the mechanical properties of different polymer electrolytes. In this study, the effects of different external factors and intrinsic electrolyte properties on the determination of the LCD are analyzed.

**Figure 5:**
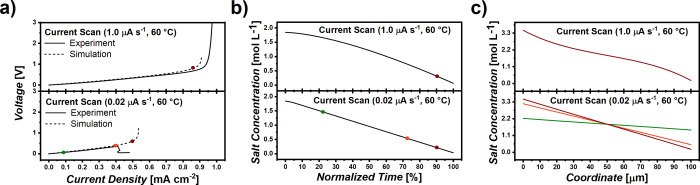
COMSOL simulated current sweep experiment. Colored points mark different times at which space-resolved concentration profiles were exported: (a) Comparison of experimental and COMSOL simulated voltage plots at fast (upper part) and slow (lower part) sweep rates. (b) Salt concentration at electrode–electrolyte interfaces, where lithium plating takes place as a function of time (to compare both experiments, the time was normalized to the cutoff time of the experiments). (c) Salt concentration as a function of cross-section coordinate (marked points in time in (a, b), reflecting the colors of the curves).

To further correlate the theory to the observed voltage behavior in the actual cells, we explored COMSOL simulated concentration gradients corresponding to the voltage profiles shown in Figure 5a. Figure 5b displays the predicted salt concentration at electrode-**|**electrolyte interfaces, where lithium plating occurs, as a function of time. Since the time necessary for such experiments is strongly dependent on the applied sweep rates, the time in the graph was normalized to the time where the cutoff time of the simulated experiment is reached. Thus, 0% refers to the start of the experiment and 100% refers to a point in time, at which the selected cutoff criterion (charge carrier depletion indicated by a steep voltage increase up to 5 V) was achieved. The concentration profile under the condition of a fast sweep rate exhibits a nonlinear decreasing behavior with a pronounced concentration gradient forming over time. In contrast, the concentration profile in the system exposed to a slow sweep rate linearly decreases, reflecting an almost constant concentration gradient. The colored points in Figure 5a,b mark different points in time, at which space-resolved concentration profiles as a function of the coordinate of the cross-section were additionally exported (Figure 5c). A coordinate of 0 μm refers to the electrode-**|**electrolyte interface where lithium metal is stripped (that is, electrochemically dissolved), while a coordinate of 100 μm refers to the corresponding electrode-**|**electrolyte interface where lithium metal is plated under the set operating conditions.

Table 1 summarizes the conditions under which such space-resolved concentration profiles were extracted, as represented by the different colors in Figure 5. When varying the cell voltage, the concentration of lithium ions at the electrode surface also changes. Interestingly, when comparing the line profile, at fast sweep rates, the space-resolved concentration profile, just before the steep voltage increase sets in (**entry 1**, Table 1), exhibits a sigmoidal behavior, resulting in a larger concentration gradient near the electrodes. In comparison, for the reduced sweep rates the curve progression is linear, indicating an almost constant concentration gradient. Hence, a faster sweep rate causes a larger concentration gradient to appear near the electrodes, also raising the peak voltage and the LCD. Moreover, when comparing the point in time just before the steep voltage increase occurs (**entry 1** and **2**, Table 1), it is observed that ion depletion appears at the surface of the electrode, where lithium plating takes place. This was monitored for both voltage plots, either at fast or slow sweep rates, thereby corroborating that the presence of the steep voltage increase can be understood as an indicator of actual charge carrier depletion, and thus may be exploited to estimate the corresponding LCDs. This seems not to be the case for the point in time just before the cell was short-circuited in the experiments with a slow sweep rate (**entry 3**, Table 1). Note that the ion concentration at the electrode-**|**electrolyte interface is decreased, but sufficient transport of ions to the electrode surface remains. Here, the actual cell short circuit is not a consequence of limited ion transport resulting in ion depletion but rather a consequence of inhomogeneous lithium plating and formation of protrusions that cause rupture of the electrolyte membranes. Also, upon comparison of the space-resolved concentration profiles at similar amounts of plated lithium metal (**entry 1** and **4**, Table 1), it can be observed that when comparable amounts of charge have passed through the cells, in the simulated experiments at a fast sweep rate, ion depletion has occurred, whereas in experiments at a slow sweep rate, the concentration profile has not changed substantially.

**Table 1:** Overview of the Different Points in Time at Which Space-Resolved Concentration Profiles Were Extracted from the Simulated Voltage Curves

entry	color code (Figure 5)	capacity [mAh]	time [h]	normalized time [%]
**1**	red (upper part, fast sweep rate)	**0.21**	0.3	**93**
**2**	red (lower part, slow sweep rate)	3.0	9.2	**93**
**3**	orange (lower part, slow sweep rate)	2.8	8.7	88
**4**	green (lower part, slow sweep rate)	**0.21**	2.3	22

In order to evaluate whether the difference between simulation and experiment at low sweep rates arises from uncertainties in determining charge carrier transport properties, a sensitivity analysis of the simulation input parameters (ionic conductivity σ_SPE_, diffusion coefficient *D*_SPE_, transference number *t*_+_, and electrode-**|**electrolyte interface resistance *R*_film_) was conducted. Sensitivity analysis quantifies how changes in model input parameters affect model outputs, helping to identify which parameters most strongly impact simulation results. In complex, highly parametrized models, this analysis guides parameter estimation by revealing the conditions and parameters that most critically determine output accuracy.^43^ The results of the analysis are listed in Figure 6. Among all of the input parameters, meaningful shifts in the simulated LCD are only observed when varying the diffusion coefficient by ±10%, both at low and high sweep rates. This shift is less pronounced at high sweep rates (Figure 6b) with a ±4% spread. However, at slower sweep rates (Figure 6a), the model output revealed increased sensitivity to a change in this parameter with a ±10% spread, yet this spread still cannot account for the experimentally observed early short circuits. These results highlight that, while LCDs determined with fast sweep rates track Sand-like transport limitations, rather slow sweep rates are more susceptible to both parameter uncertainty and mechanical breakdown of separators, emphasizing the importance of validating LCD measurements under various experimental conditions.

**Figure 6:**
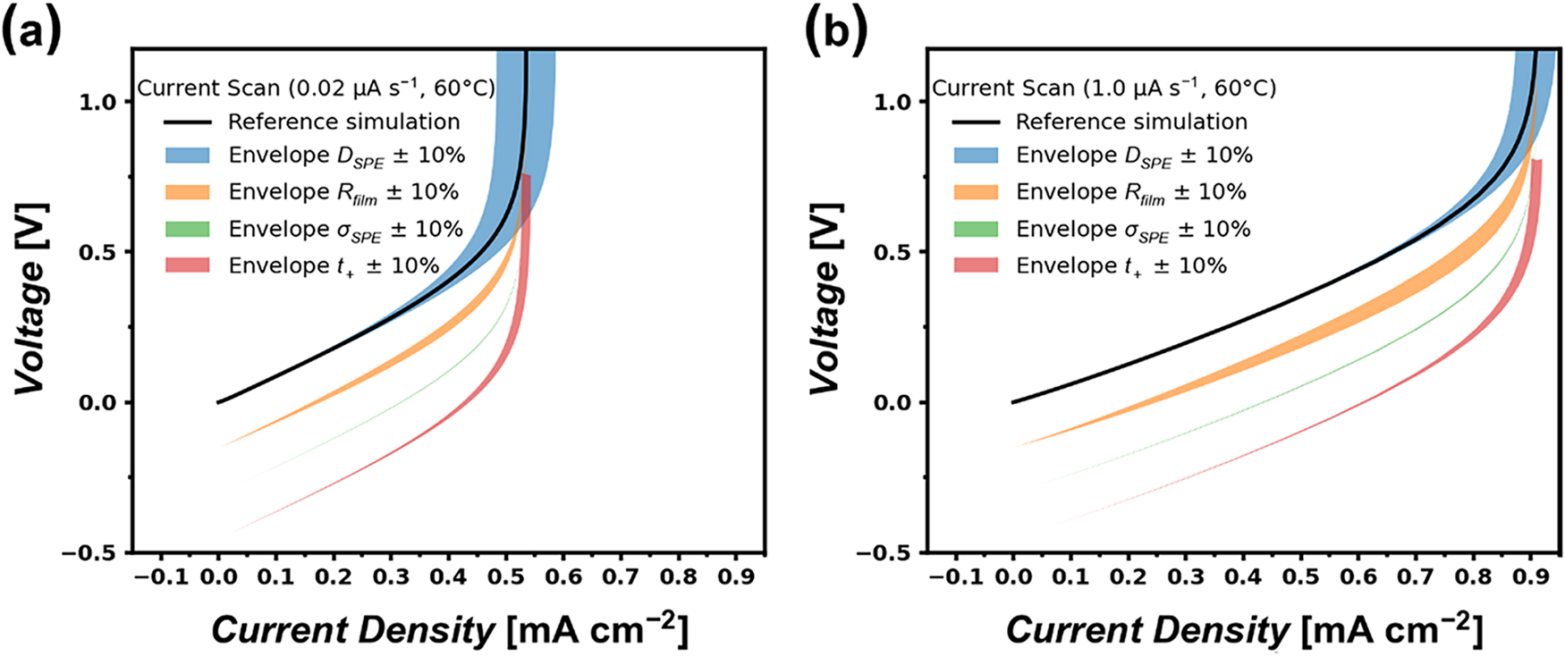
Sensitivity analysis for several simulation input parameters shows ±10% envelopes. Individual envelopes are shifted downward for visual clarity. (a) Sensitivity envelopes for 1.0 μA s^–1^ sweep rate. (b) Sensitivity envelopes for 0.02 μA s^–1^ sweep rate.

Since simulated and experimental data are not in agreement when applying a slow current sweep at 60 °C in the case of cells with cross-linked PEO membranes, additional characteristics of the considered materials might come into play that vary the obtained LCDs. Therefore, various PEO-based electrolytes with individually adjusted moduli were examined. According to the literature, PEO-based electrolytes allow for limited lithium protrusions if the membranes provide shear moduli on the order of *G* ^SPE^ > 10^–3^ *G* ^Li^ (∼3 MPa).^44^ Approaches to boost the mechanical property of polymer electrolytes include (1) chemical cross-linking to produce denser networks,^45,46^ (2) filling or infiltration of ceramic nanoparticles,^47,48^ and (3) a design of suitable block copolymers with mechanically more robust blocks.^23^ Indeed, the ionic conductivity and resulting morphology of the prepared block copolymers are summarized in Figure S1. Note that in previous work, we discussed ion transport within the considered nanophase separated block copolymers in detail.^23,49^ Figure 7 displays data of mechanical properties of the different electrolytes and the resulting values of limiting current densities. Figure 7a includes the loss and storage moduli of the different solid polymer electrolytes derived from rheological measurements. Linear and cross-linked PEO exhibit similar mechanical stabilities with moduli in the range of 0.1 MPa, while infiltration of ceramic particles somewhat enhances the mechanical stability of linear PEO. Upon introduction of a mechanically more stable polymer block (such as styrene), the overall modulus is increased, yielding ∼ 1.0 MPa. It should be mentioned that the actual derived shear modulus represents a mean value over the electrode area. Corresponding AFM phase images (included in Figure S1c,d) clearly visualize the nanophase separation of the different phases, evidencing a typical Turing pattern reflecting a distribution of hard and soft polymer phases within the polymer membranes. Pristine polystyrene can withstand mechanical stress up to the GPa range^50^ and could nominally limit lithium dendritic protrusions, while Figure 7b demonstrates long-term single-side lithium deposition, thereby corroborating the ability of mechanically reinforced block copolymers to withstand dendritic protrusions for ∼100 h. In contrast, in the case of electrolytes based on linear PEO, a cell short circuit after a few hours was observed.

**Figure 7:**
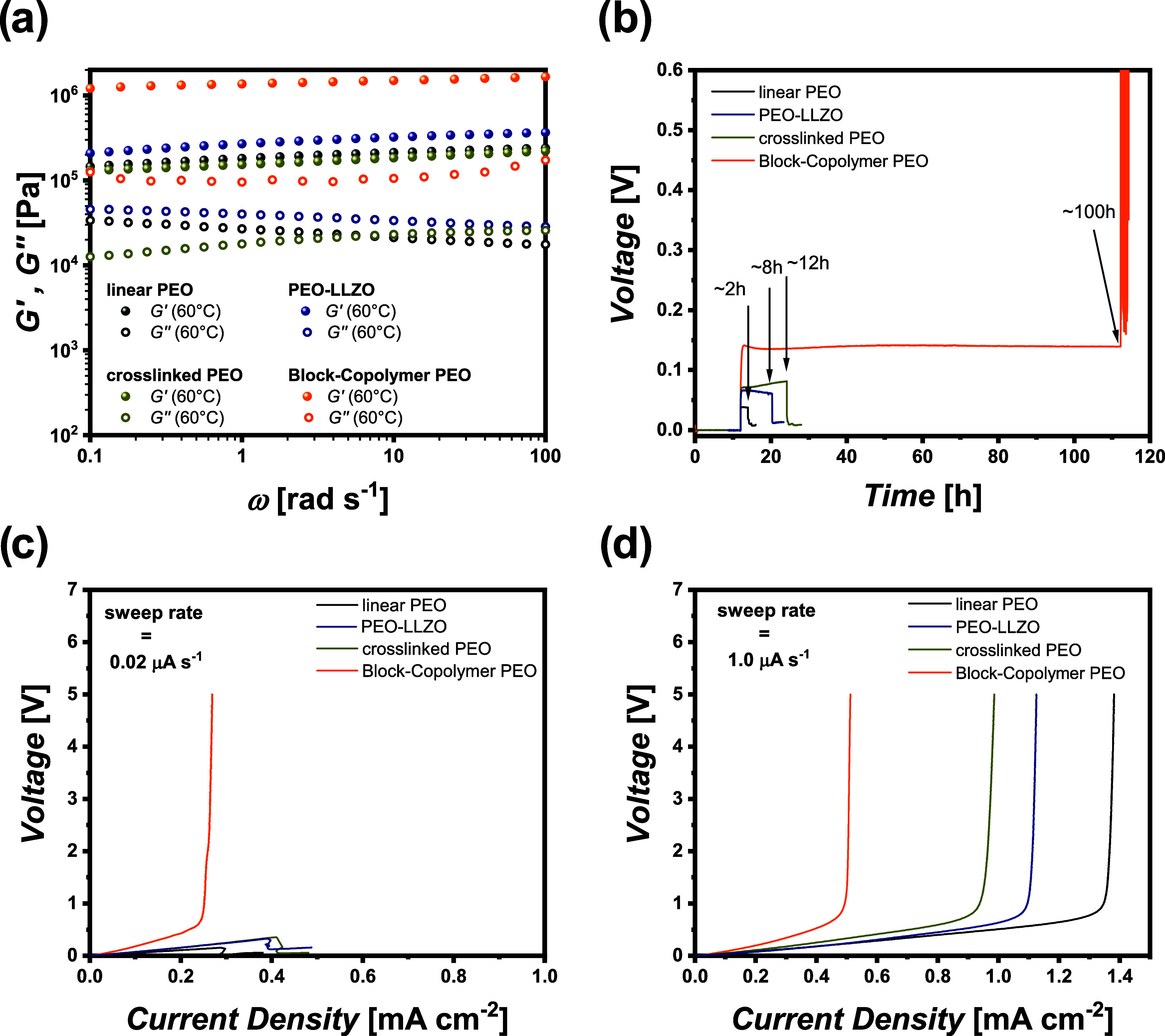
Mechanical properties of selected PEO-based polymer electrolytes. (a) Rheology data, (b) single-side lithium deposition experiment at a current density of 0.1 mA cm^–2^, and (c) current scans at slow (0.02 μA s^–1^) and (d) fast (1.0 μA s^–1^) sweep rates, upon operating cells with various PEO-based polymer electrolytes.

A current scan at a sweep rate of 0.02 μA s^–1^ (Figure 7c) as well as 1 μA s^–1^ (Figure 7d), both at 60 °C, was conducted for all four prepared model-type PEO-based electrolytes. Note that a slow sweep rate also means prolonged period of lithium deposition on the same electrode. Similar to Figure 5 and Table 1, the data are depicted in the Supporting Information for the various PEO-based electrolytes (Figure S2 as well as Tables S1–S4). At slow sweep rates, the current density at which the same amount of lithium was deposited compared to the experiment at fast sweep rate is highly dependent on the electrolyte’s properties (ion transport as well as mechanical properties), but no significant deviation was observed. In good agreement with the constant current experiments mentioned before (see Figure 7b), the dynamic current scan at a slow sweep rate shows a sudden cell short circuit in the case of electrolytes based on linear PEO. Solely the mechanically reinforced block copolymer mitigated the occurrence of lithium protrusions induced by inhomogeneous lithium deposition sufficiently long enough to reach the typical concentration polarization (up to the 5.0 V cutoff conditions) that results from transport limitations and ion depletion (Figure 7c). This demonstrates that dendritic lithium growth and subsequent failure are not inevitable but rather depend critically on the applied protocol, operating conditions, and mechanical stability of the electrolytes, as reflected by rheology data (Figure 7a). Dendrite formation mechanisms across the studied PEO-based electrolytes are not assumed to be identical. Instead, differences in mechanical stability and interfacial response determine how lithium growth propagates and whether cell failure occurs based on short-circuiting or transport-limited polarization. Electrochemical impedance measurements (EIS) were conducted before and after the current scans (Figure S5 and Table S8) to elucidate different failure mechanisms of the cells. The impedance spectra before the current scans show a typical semicircle, reflecting electrolyte’s resistances, in good agreement with already extracted ionic conductivity data (Figure S1a). After the current scan, the EIS spectra differ in the magnitude of the impedances and the shape of the Nyquist plot. For the electrolytes based on linear PEO, the semicircle is shifted to nearly 0 Ω cm^2^, which is attributed to the impedance of lithium protrusions, causing a rupture of the electrolyte membranes and short circuit of the cell. However, the impedance of the block copolymer is increased, mainly due to the occurrence of ion depletion, which eventually results in altered electrode-**|**electrolyte interfaces and consequently changed resistances. This condition in practice yields a steep increase in the voltage, in this way compensating for insufficient ion diffusion, and finally affords lithium dendrite growth and cell failure. In contrast, at fast sweep rates, all of the electrolytes suffer from concentration polarization due to ion depletion. Here, the determined LCD values strictly follow the behavior of ionic conductivity of the electrolytes. A higher ionic conductivity provides improved mobility of lithium ions within the electrolyte, which shifts the condition of likely ion depletion to appear at higher currents. Note that electrolytes made from linear PEO exhibit highest ionic conductivity and LCD values at the cell operating temperature of 60 °C, whereas block copolymer-based membranes have reduced ionic conductivity and thus tolerate smaller LCD values. Nevertheless, the block copolymer is able to suppress the height of lithium protrusion by plastic deformation due to its superior mechanical properties, and is therefore able to withstand inhomogeneous lithium deposition, enabling determination of the LCD. Another crucial factor, which should be considered when determining the limiting current densities, comprises the cell temperature. The latter not only governs the achievable ionic conductivity (see Supporting Information and Figure S1a) but also limits the mechanical properties of the electrolytes.

Though an elevated cell operating temperature accelerates ion movement within the electrolytes, it also weakens the mechanical stability of the polymer membranes. Exemplarily, Figure 8a shows the temperature dependence of shear moduli of cross-linked PEO membranes, indicating softening of the mechanical stability while increasing the temperature. Likewise, Figure 8b,c display data from current scans at slow and fast sweep rates at different temperatures. At reduced sweep rates, a reduction of the cell temperature from 60 to 40 °C already yields the characteristic voltage behavior in the case of ion depletion and the associated concentration polarization, whereas the current scan at 60 °C suffers from weak mechanical stability and a sudden short circuit due to electrolyte rupture. Note, however, that the temperature itself has an impact on the LCDs (Figure 8b,c). Finally, the impact of stack pressure was also considered. Especially when operating cells with solid-state electrolytes, the stack pressure appears to be an underestimated factor, despite the fact that any interfacial issues among electrolytes and electrodes become even more important, ultimately governing cell longevity.^10^

**Figure 8:**
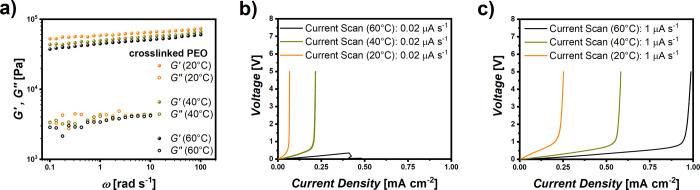
Effect of cell operating temperature on the determination of LCDs in the case of cross-linked PEO electrolytes. (a) Mechanical stability of cross-linked PEO at 20 °C, 40 °C, and 60 °C (b) current scan at slow and (c) increased sweep rates at 20 °C, 40 °C, and 60 °C.

For ceramic-based solid electrolytes such as oxides or sulfides, it is well established that higher external pressures (up to hundreds of bar)^51^ improve the overall electrochemical cell performance due to better electrode-**|**electrolyte contacts.^52,53^ Due to the superior wettability and flexibility of polymer-based solid electrolytes, applied external pressure could be considerably lower. However, the impact of externally applied pressure is often underestimated in polymer-based solid-state batteries, and limited data is available that illustrates the effects of stack pressure on the polymer properties.^54,55^ Moreover, compression of electrolyte membranes due to the flexible character of polymers should be considered, which is often tedious to determine, making it even harder to clearly investigate the impact of the applied pressure. Previously, we analyzed the impact of external pressure on the pouch cell performances of different polymer electrolytes. Mechanical investigations reflected that compressive elastic deformation appears for solid polymer electrolytes, and starting from a threshold value, irreversible plastic deformation occurred. Below this threshold value, stable electrochemical cycling was observed. Notably, this condition is strongly dependent on the actual polymer and should be carefully determined.^55^ Besides, in the reported literature, it is often discussed that the thickness of lithium metal electrodes plays an essential role for boosting cell longevity and overall performance.^56^ However, it has to be taken into account that when replacing a thick lithium foil by a thinner lithium foil the stack pressure might also be altered. Note that the pressure in the coin cells is adjusted by the thickness of the spacer and the absolute height of the cell stack. When the lithium metal foil thickness is reduced, a thicker spacer has to be used to maintain the same pressure in the cell. Slight changes in the thickness can have a significant impact on the stack pressure. Thus, both effects were explored here for two cases, either maintaining the stack pressure but invoking variable thickness of lithium metal foils or keeping lithium thickness but alternating the stack pressures. Consequently, Figure 9a shows the obtained limiting current densities for Li|cross-linked PEO|Li cells with different thicknesses of the lithium foil, where the stack pressure is maintained at 1.88 bar. The thickness of lithium metal discs does not notably vary the resulting LCD, whereas the stack pressure (1.88 or 5.65 bar) has a meaningful effect on the LCD (Figure 9b). At higher stack pressure, the contacts between the electrolyte and electrode are also improved, generally affording more homogeneous lithium deposition and especially current distribution.^10^ Poor interfacial contacts could yield local current peaks that potentially exceed the actual LCD, thereby fostering local charge carrier depletion and lithium dendrite growth. Note that the experimentally examined LCD denotes an averaged value over the electrode area, assuming ideal contacts between the electrolyte and electrode. At a stack pressure of 1.88 bar, the respective LCD amounts to 0.96 ± 0.02 mA cm^–2^, whereas at a stack pressure of 5.65 bar, the LCD is already increased to 1.06 ± 0.04 mA cm^–2^ (Figure 9b). We investigated these effects in full cells in detail in our previous study.^55^ Based on these results, the applied stack pressure is in a reasonable range for polymer-based lithium metal batteries.

Especially when striving for higher mass loadings of cathode active materials in an industrially relevant solid-state battery setup, the charge that is going to pass the electrolyte will also increase. Hence, it is important not only to figure out the LCD by using comparatively short dynamic experiments with insignificant amounts of deposited lithium metal but also to cross-check data by performing more realistic galvanostatic experiments. Typically, lithium plating and stripping experiments in Li||Li cells consist of periodical deposition followed by stripping of comparable amounts of lithium (1 mA cm^–2^ represents 5 μm of Li). In principle, by increasing the applied current density stepwise and cell cycling for a defined number of cycles prior to increasing the actual current density again, it is also feasible to derive the maximum endurable current density, as reflected by the condition of either a sudden voltage drop or appearance of cell concentration polarization.^10,29^

**Figure 9:**
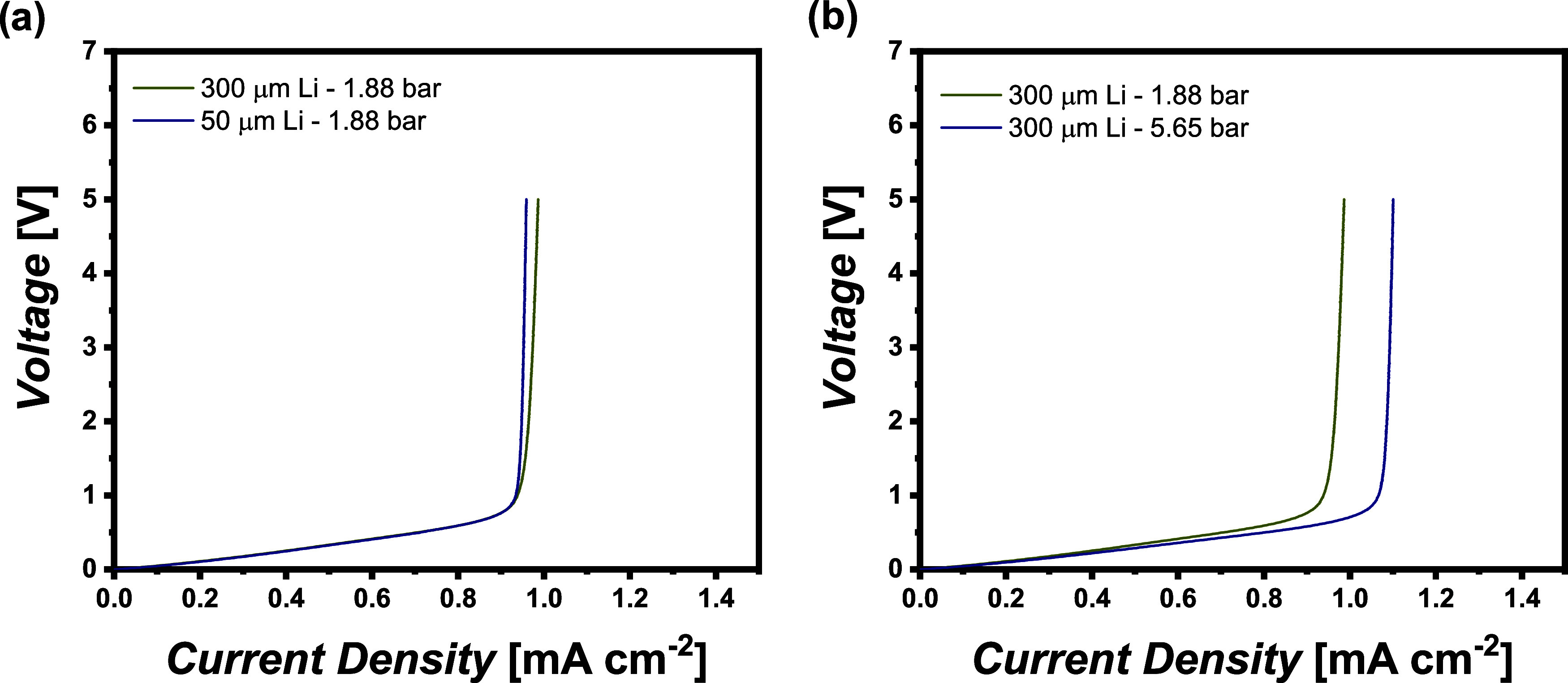
Galvano-dynamic determination (sweep rate of 1 μA s^–1^) of the LCD of cross-linked PEO at different stack pressures. (a) Thickness of utilized lithium metal foils while maintaining the stack pressure; (b) different stack pressures while maintaining the thickness of lithium.

Note that the utilized parameters such as the number of repeated cycles or the mode (time-controlled or capacity-controlled) may also vary the obtained results. Figure 10 exhibits the result of capacity-controlled galvanostatic experiments with alternating current densities, where the capacity is fixed to either 0.4 mAh cm^–2^ (Figure 10a) or 1.6 mAh cm^–2^ (Figure 10b), in this way considering potentially increased cathode mass loadings. Note that at a current density of 0.1 mA cm^–2^, lithium metal is continuously plated for a duration of 4 h to afford a fixed capacity of 0.4 mAh cm^–2^, whereas lithium metal is plated over 16 h to yield a fixed capacity of 1.6 mAh cm^–2^. Interestingly, the results derived from the galvanostatic experiments are not in good agreement with the values extracted by dynamic experiments, accounting for the fact that merely the mechanically more robust block copolymer withstands lithium protrusion in both cases, either at a fixed capacity of 0.4 mAh cm^–2^ or 1.6 mAh cm^–2^. In the latter case, all of the cells with electrolytes based on linear PEO suffer from short circuits after a few hours within the first plating step (see Figure 10b), whereas in the case of fixed capacity of 0.4 mAh cm^–2^, LCDs of cells with block copolymer as well as cross-linked PEO corroborate the limiting current densities established from the dynamic sweep experiments. The linear PEO and PEO-LLZO do not reflect the comparably high LCDs resulting from the dynamic experiments (Figure 10a). This is supported by full cell cycling data available in the literature, emphasizing rapidly decomposing polymer electrolytes if based on unmodified, linear PEO, often yielding voltage noise and cell short circuits.^45,46^ In fact, this observation corroborates the necessity to always cross-check LCD data derived from dynamic experiments, strengthening the consideration of the mechanical stability of electrolytes to not overestimate the determined LCD data.

**Figure 10:**
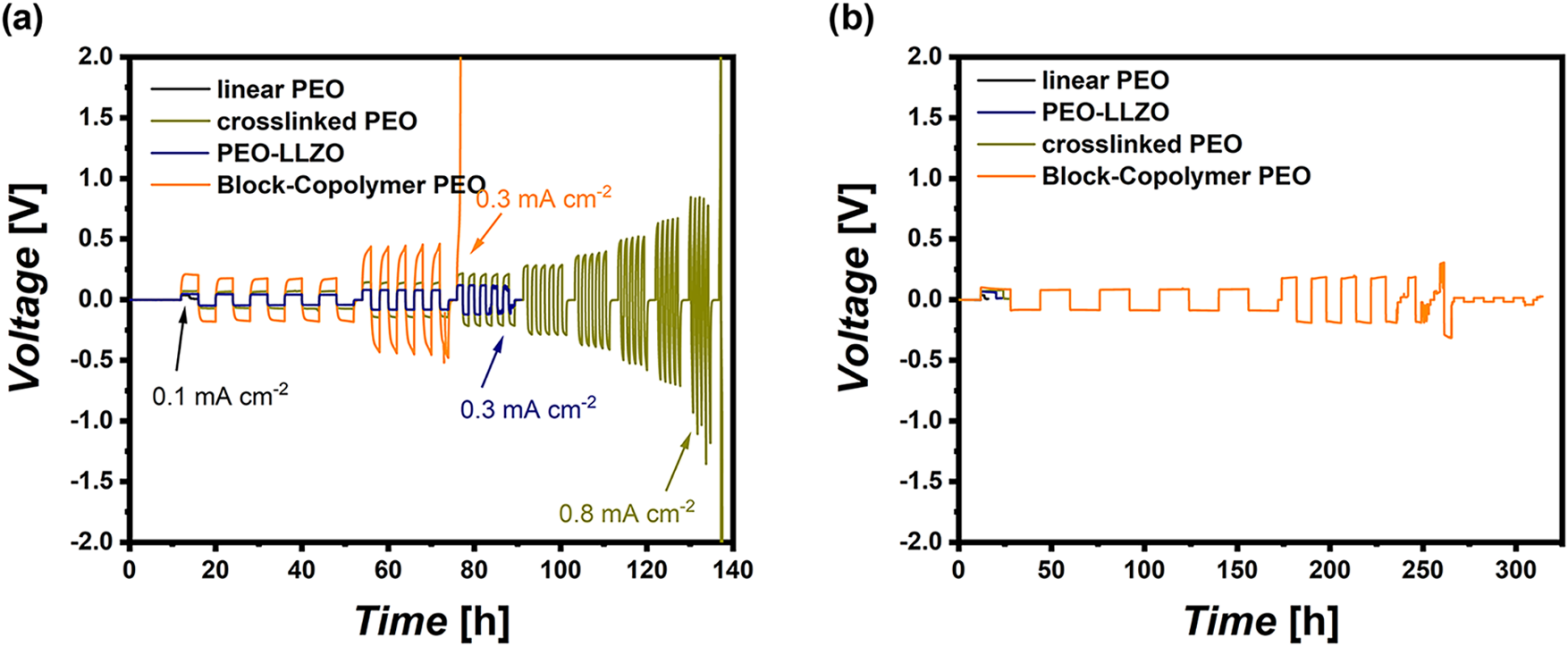
Galvanostatic experiments of Li||Li cells operating on different PEO-based electrolytes. Capacity-controlled plating and stripping experiments at an alternating current density until a fixed capacity of (a) 0.4 mAh cm^–2^ or (b) 1.6 mAh cm^–2^ was achieved.

## Conclusion

In this work, we explored different experimental procedures to derive the limiting current density of cells operating on solid polymer electrolytes. The applied sweep rates as well as temperature and stack pressure were systematically varied, and the effect on the results was critically evaluated and compared with data extracted from COMSOL simulations. Notably, we determined limiting current densities for cross-linked PEO to be in the range of 0.4–1.0 mA cm^–2^, strongly depending on the selected measurement parameters but within typical ranges for such solid polymer electrolytes.^28^ Actually, the mechanical properties of polymer electrolytes play an essential role in interpreting estimated values of the limiting current density, as underlined by the simulations. These COMSOL simulations take only transport properties within electrolytes into account, neglecting mechanical features, and in the case of high sweep rates, both simulation and experiments are in good agreement. However, at low sweep rates, discrepancies between simulation and experiment were observed, corroborating that the experiment does not suffer from ion transport limitations but rather from mechanical issues within polymer electrolytes. To support this interpretation, a sensitivity analysis of simulation input parameters revealed that while LCD predictions at low sweep rates are more sensitive to deviations in transport properties, the observed discrepancies still cannot be reconciled without invoking mechanical failure. This confirms that transport limitations alone are insufficient to explain early cell failure under slow sweep conditions. Cell failure due to eventual rupture of polymer membranes caused by local lithium protrusions and inhomogeneous lithium deposition was experimentally reflected by sudden voltage drops, displaying cell short circuits. Hence, mechanically reinforced polymer electrolytes may sufficiently limit lithium protrusions, so that for faster charge applications, polymer electrolytes should ideally allow for high ionic conductivity to inhibit ion depletion at electrode–|electrolyte interfaces as well as superior mechanical properties to compensate for potentially uneven lithium deposition. Invoking high sweep rates in dynamic experiments, the derived values of limiting current densities strictly follow the ionic conductivity of the investigated model-type polymers, as suggested by Sand’s model. However, application of lower sweep rates may induce mechanical stress of polymer electrolytes by lithium protrusion, e.g., due to inhomogeneous lithium deposition, eventually yielding a sudden cell short circuit upon membrane rupture. Therefore, the experimentally established maximum endurable current density, which could be applied without causing cell failures, does not necessarily reflect LCDs predicted by Sand’s model, especially in cases where cells suffer from mechanical weakness of the polymer electrolytes. Moreover, invoking cyclovoltammetry at different sweep rates demonstrates that the observable peak current is proportional to the square root of the sweep rate (as indicated by the Randles–Sevcik equation). Using a single sweep rate for the determination of limiting current densities could potentially result in under- or overestimated values for the LCDs. External parameters such as temperature and stack pressure also strongly affect the actual limiting current density, where a trade-off between higher ion mobility, limited mechanical strength, and plastic deformation at higher temperatures is required in practice. External pressure applied to the cell stack potentially improves the tolerated limiting current densities, attributed to enhanced contacts at (lithium) electrode-**|**electrolyte interfaces that foster more homogeneous lithium deposition as well as (local) current distribution, as long as pronounced membrane compression or even thinning can be avoided.^55^ In summary, the experimentally determined limiting current densities appear to not reflect intrinsic key performance indicators of polymer electrolytes but rather are affected by (often ignored) external factors, thereby balancing conflicting demands that have to be carefully examined to render polymer electrolytes suitable for application in high-performance lithium metal cells. To this end, we strongly recommend conducting dynamic experiments with at least one sweep rate far below and one above the estimated threshold value ν_lim_ (polymer-specific, 0.3 μA s^–1^ for cross-linked PEO at 60 °C). A fast sweep rate mirrors the limiting current density according to Sand’s model, whereas a slow sweep rate displays the polymer electrolyte’s ability to mitigate lithium protrusions, depending on the ion transport properties and mechanical stability of the considered polymer electrolytes. Finally, galvanostatic experiments that reflect more realistic battery cell conditions should also be executed and compared with data extracted from dynamic experiments. In doing so, processes that are unfavorably limiting the current flow required for fast charge applications could systematically be analyzed to provide pathways for tailored materials optimization.

## Supplementary Material



## Notes

Calculation of the cell stack pressure, simulation details, ion transport and nanophase separation, current sweep experiments with different electrolytes, OCV data, and impedance spectra (PDF)

## Abbreviations

LCD: limiting current density
LSV: linear sweep voltammetry
CV: cyclic voltammetry
CS: current scan
PEO: poly-ethylene oxide
BP: benzophenone
LiTFSI: bis­(trifluoromethane)­sulfonimide lithium salt
LLZO: lithium lanthanum zirconium oxide (Li_7_La_3_Zr_2_O_12_)
UV: ultraviolet
