# Supplementary material for: A Critical Evaluation of the Limiting Current Density in Polymer Electrolytes: Interplay of Ion Transport, Mechanical Stability, and Conformal Li–Electrolyte Interfaces
Source: J Am Chem Soc. 2026 Jan 23;148(4):4176–88. doi: 10.1021/jacs.5c16267 (PMC12879923; doi:10.1021/jacs.5c16267)
Supplement: Supplementary file 1 [file ja5c16267_si_001.pdf]

Supporting Information

A Critical Evaluation of the Limiting Current Density in Polymer Electrolytes – Interplay of Ion Transport, Mechanical Stability and Conformal Li-Electrolyte Interfaces

Philipp Röring[#,a], Jan Pleie[#,a,b,c], Andreas J. Butzelaar[d], Gerrit M. Overhoff[a], Christina Schmidt[a], Kerstin Neuhaus[a], Patrick Théato[d,e], Martin Winter[a,b,c], Gunther Brunklaus[a,b]*

a) Forschungszentrum Jülich GmbH, Insitute for Energy Materials and Devices 4 (IMD-4), Helmholtz-Institute Münster (HI MS), Corrensstraße 46, 48149, Münster, Germany.

b) University of Münster, Institute of Physical Chemistry / MEET Battery Research Center, Corrensstraße 46, 48149 Münster, Germany.

c) University of Münster, International Graduate School for Battery Chemistry, Characterization, Analysis, Recycling and Application (BACCARA), Corrensstr. 40, 48149 Münster, Germany.

d) Karlsruhe Institute of Technology (KIT), Institute for Chemical Technology and Polymer Chemistry (ITCP), Engesserstraße 18, 76131 Karlsruhe, Germany.

e) Karlsruhe Institute of Technology (KIT), Soft Matter Laboratory - Institute for Biological Interfaces III (IBG-3), Hermann-von-Helmholtz-Platz 1, 76344 Eggenstein-Leopoldshafen, Germany.

# These authors contributed equally.

Email: g.brunklaus@fz-juelich.de

Table of Contents

Calculation of the cell stack pressure 2
Simulations 4
Ion transport and morphology of the block copolymer 10
Current sweep experiments with different sweep rates 15
Open circuit voltage (OCV) during conditioning-step 17
Electrochemical impedance spectroscopy 18
References 21

Calculation of the cell stack pressure

According to **equation (1)** the pressure p can be calculated,

$$p = \frac{F}{A} = \frac{D \cdot \Delta L}{A} \tag{1}$$

where F is the force and A is the area on which the force is applied. The force can be calculated by using the spring constant D ($D = 100\ \mathrm{N\ mm^{-1}}$) and the deflection of the spring ΔL. D was measured by using a Zw*i*ck Roell spring testing machine. Therefore, a force of $F = 50\ \mathrm{N}$ was applied and the deflection ΔL of the spring was measured. The deflection was $\Delta L = 0.5\ \mathrm{mm}$ as an average of ten measurements. According to **equation (2)**, the spring constant was calculated to be $D = 100\ \mathrm{N\ mm^{-1}}$.

$$D = \frac{F}{\Delta L} \tag{2}$$

ΔL of the spring in the crimped cell was calculated according to **equation (3)** by subtracting the thickness of the individual cell components (**Table S1**) from the thickness of the crimped cell being $h_{\mathrm{cell}} = 3.2\ \mathrm{mm}$ for CR2032-type cells.

$$|\Delta L| = h_{cell} - (h_{case} + h_{spacer} + h_{an.} + h_{SPE} + h_{cat.} + h_{spacer} + h_{spring} + h_{case}) \quad (3)$$

Thus, by using a lower spacer with a thickness of 0.5 mm or 1.0 mm a cell stack pressure of 1.88 bar or 5.65 bar was calculated, respectively.

Table S1: The thickness of the different cell components in order to calculate the deflection of the spring when the cell is crimped.

**Individual cell component**	***L* (thickness) [mm]**
lower case	0.25
lower spacer	0.50 / 1.00
anode	0.30
solid polymer electrolyte	0.10
cathode	0.30
upper spacer	0.50
spring	1.25
upper case	0.25

Simulations

The simulations are based on the theory of concentrated solutions developed by John S. Newman and others.[1] The software COMSOL Multiphysics 6.0 is used to model the Li||Li cells, where the lithium metal electrodes are described as two-dimensional surfaces that function as both ion source and ion sink.

Electrode surfaces:

At the interfaces of the lithium metal and the electrolyte, charge transfer reactions are modeled using the Butler-Volmer-equation:

$$i_{\mathrm{loc}} = i_0 \left(\exp\left(\frac{\alpha F \eta}{RT}\right) - \exp\left(-\frac{(1-\alpha) F \eta}{RT}\right) \right) \quad (4)$$

where i_0 is the exchange current density, α is the anodic transfer coefficient (also called the symmetry factor), F is Faraday's constant, R is the gas constant, T is the temperature in Kelvin, and η is the overpotential. The exchange current density is dependent on the concentration at the electrode surface:

$$i_0 = i_{0,\mathrm{ref}} \left(\frac{c_{\mathrm{SPE}}}{c_{\mathrm{SPE,ref}}} \right)^{\alpha} \quad (5)$$

where $i_{0,ref}$ is the reference exchange current density at the reference electrolyte salt concentration $c_{SPE,ref}$ and c_{SPE} is the actual salt concentration. The overpotential η is given as

$$\eta = \phi_{\mathrm{s,ext}} - \Delta\phi_{\mathrm{s,film}} - \phi_{\mathrm{SPE}} - E_{\mathrm{eq}} \quad (6)$$

where $\phi_{s,ext}$ is the externally applied potential, $\Delta\phi_{s,film}$ is the potential drop across a film resistance R_{film} of the electrode, e.g. caused by an SEI, ϕ_{SPE} is the electrolyte potential at the electrodes surface and E_{eq} is the equilibrium potential of the electrode reaction. The potential drop across the film on the electrodes is determined by the product of a film resistance R_{film} and the total current density through the film:

$$\Delta\phi_{\mathrm{s,film}} = R_{\mathrm{film}} \cdot i_{\mathrm{total}} \tag{7}$$

Electrolyte:

The scalar current density i_{loc} is related to the current density vector in the electrolyte $\vec{i}_{\mathrm{SPE}}$ using the normal vector of the surface $\vec{n}$

$$\vec{n} \cdot \vec{i}_{\mathrm{SPE}} = i_{\mathrm{loc}} \tag{8}$$

And the total current at the electrode surface $I_{SPE,total}$ is determined via the surface integral

$$\int_{\partial A} \vec{i}_{\mathrm{SPE}} \cdot \vec{\mathrm{n}}\,\mathrm{d}l = I_{\mathrm{SPE,total}} \tag{9}$$

The model describes the transport of ions through the polymer electrolyte by migration, i.e. the transport of charged particles caused by the presence of an electric field, and by diffusion, i.e. the transport of particles caused by a concentration gradient. Convection is neglected in this approach, as a solid polymer electrolyte is used in the cells.[2] In the electrolyte, the first set of equations ensures the conservation of current according to

$$\nabla \cdot \vec{i}_{\mathrm{SPE}} = 0 \tag{10}$$

$$\vec{i}_{\mathrm{SPE}} = -\sigma_{\mathrm{SPE}} \cdot \nabla \cdot \phi_{\mathrm{SPE}} + \frac{2\sigma_{\mathrm{SPE}} RT}{F} \cdot \left(1 + \frac{\partial \ln f}{\partial \ln c_{\mathrm{SPE}}}\right) \cdot (1 - t_{+}) \cdot \nabla \cdot \ln c_{\mathrm{SPE}} \tag{11}$$

where σ_{SPE} is the electrolyte ionic conductivity, f is the activity coefficient of the salt and t_{+} is the transference number. The second set of equations solved for governs the mass balance of the electrolyte salt

$$\frac{\partial c_{\mathrm{SPE}}}{\partial t} + \nabla \cdot \vec{J}_{\mathrm{SPE}} = R_{\mathrm{SPE}} \tag{12}$$

$$\vec{J}_{\mathrm{SPE}} = -D_{\mathrm{SPE}} \cdot \nabla \cdot c_{\mathrm{SPE}} + \frac{\vec{i}_{\mathrm{SPE}} \cdot t_{+}}{F} \tag{13}$$

where $\vec{J}_{SPE}$ is the flux of ions, R_{SPE} is the reaction rate, which is non-zero at the electrode surfaces and D_{SPE} is the diffusion coefficient of the electrolyte salt.

The diffusion coefficient of the electrolyte can be expressed as

$$D_{\mathrm{SPE}} = D_{0,\mathrm{SPE}} \cdot \left(1 + \frac{\partial \ln f}{\partial \ln c_{\mathrm{SPE}}}\right) \tag{14}$$

Here $D_{0,SPE}$ is the self-diffusion coefficient of the electrolyte and the term in brackets $\left(1 + \frac{\partial \ln f}{\partial \ln c_{\mathrm{SPE}}}\right)$ is referred to as the thermodynamic factor.

Additional boundary conditions are applied at the surfaces of the electrolyte, i.e. the no-ion-flux boundary condition

$$-\vec{n} \cdot \vec{J}_{\mathrm{SPE}} = 0 \quad (15)$$

on all surfaces as well as the zero-current boundary condition

$$-\vec{n} \cdot \vec{i}_{\mathrm{SPE}} = 0 \quad (16)$$

on the surfaces which do not interface with the electrodes.

Cylindrical symmetry:

Since coin cells essentially show cylindrical symmetry, the COMSOL software can utilize this symmetry to represent a three-dimensional cartesian geometry within a cylindrical coordinate system. In this coordinate system, the solution varies only along two orthogonal coordinates and the system is symmetric along the third angular coordinate, substantially reducing computational complexity without sacrificing the validity of the approach.

Material parameters:

The accuracy of the COMSOL simulation results depends on the material properties supplied as input parameters for the simulation. The following table provides an overview of the material properties used.

Table S2: Material parameters of crosslinked PEO used as input parameters for the simulation.

Parameter	Symbol	Value [unit]	Comment
Reference salt concentration	$c_{SPE,ref}$	1.84 ± 0.2 [mol L^{-1}]	experimental value*[1]
Electrolyte conductivity	σ_{SPE}	$2.95 \cdot 10^{-4} \pm 9.3 \cdot 10^{-5}$, $1.08 \cdot 10^{-4} \pm 7.3 \cdot 10^{-6}$[S cm^{-1}] at 60, 40 °C	experimental value (impedance)*[2]
Transference number	t_+	$0.1328 \pm 1.35 \cdot 10^{-3}$	experimental value (PFG-NMR)*[3]
Self-diffusion coefficient	$D_{0,SPE}$	$6.77 \cdot 10^{-13}$, $1.44 \cdot 10^{-13} \pm 1.7 \cdot 10^{-15}$ [m^2 s^{-1}] at 60,40 °C	experimental value (PFG-NMR) at 40 °C*[3], fitted for 60 °C*[4]
Thermodynamic factor	$\left(1+\frac{\partial \ln f}{\partial \ln c_{SPE}}\right)$	1.7	fitted for 40 °*[4], assumption for 60 °C
Anodic transfer coefficient	α	0.5	assumption based on literature[3]
Film resistance	R_{film}	225, 450 [Ω cm^2] at 60, 40 °C	fitted
Reference exchange current density	$i_{0,ref}$	1.2 [mA cm^{-2}]	assumption based on literature[4]

*[1]The molar concentration was calculated as mean out of four membranes with known dimensions, weight and lithium salt content.

*[2]See **Figure S1a)**

*[3]The self-diffusion coefficient was determined by NMR. All the spectra were recorded with a BRUKER 4.7 T Avance III instrument using a commercial BRUKER diff50 probe. Pulsed field gradient NMR data were acquired with a (double tuned ^{7}Li, ^{1}H-^{19}F) 5 mm coil at 40 °C (±0.1 °C). A 0.25 M LiCl in H_2O and a 3 M KF in H_2O solution were utilized for external calibration. The gradient strength was set to max. 2945 G cm^{-1} averaging up to 16 scans with a gradient pulse length δ of 1 ms (^{19}F) and 2 ms (^{7}Li) and diffusion time Δ varied from 60 ms (^{19}F) to 400 ms (^{7}Li). The self-diffusion coefficients D of the lithium and fluorine species were derived from a stimulated echo sequence ("diffSte") after fitting the overall attenuated signal amplitudes to the Stejskal-Tanner equation[5,6], which describes the case of rather ideal ("free") isotropic diffusion:

$$I = I_0 \cdot e^{(-D \gamma^2 \delta^2 g^2 (\Delta - \frac{\delta}{3}))} \quad (17)$$

With I being the signal intensity, I_0 the initial signal in the absence of a magnetic field gradient and γ the gyromagnetic ratio. Data analysis was done with BRUKER Topspin 3.5pl7 and BRUKER Dynamics Center 2.5.

*[4]The thermodynamic factor at 40 °C was fitted based on the equation 14 and the self-diffusion coefficient derived from PFG-NMR experiments at 40 °C. Assuming that the thermodynamic factor remains constant upon changing the temperature to 60 °C, it was possible to fit the self-diffusion coefficient at 60 °C. The self-diffusion coefficient at 60°C was determined as the average of three separate experimental fits. Two experiments were constant-current experiments at different current densities. The third was a current sweep experiment (**Figure 2b)** and **Figure 5a)**

upper part) with a high sweep rate(1 μA s^{-1}) at which the cell did not short-circuit during the experiment.

Ion transport and morphology of the block copolymer

Measurements of the ionic conductivity

The corresponding ionic conductivities of the different SPEs were measured by electrochemical impedance spectroscopy (EIS). The samples were prepared by placing the polymer electrolyte film (ø = 13 mm) between two stainless-steel electrodes in a coin cell-type (CR2032) cell setup. To improve the interfacial contacts between electrodes and electrolyte, a preheat temperature loop was performed before cooling down the samples to 0 °C. The measurements were carried out using a Metrohm Autolab potentiostat in the temperature range between 0 °C to 70 °C. Impedance measurements were conducted over a frequency range from 1 MHz to 100 mHz with an amplitude of 10 mV. A heating cycle comprised of a gradual temperature increase in 10 °C steps from 0 °C to 70 °C; after each temperature change, the temperature was held constant for two hours before acquiring the impedance spectra. At a temperature of 70 °C, the heating profile was reversed and gradually cooled down to 0 °C in 10 °C temperature steps. The corresponding ionic conductivity σ of the SPEs was obtained according to **equation (18)**

$$\sigma = \frac{1}{R_\mathrm{b}} \cdot \frac{l}{A} \tag{18}$$

With R_b being the bulk electrolyte resistance that can be accessed from the Nyquist plot, l denotes the film thickness and A the film area.

Small-Angle X-Ray Scattering (SAXS)

The morphology of the block copolymers was studied by 2D-SAXS measurements, invoking on a Hecus S3-Micro X-ray system using equipped with a point microfocus source, 2D X-ray mirrors, and a two-dimensional CCD detector from Photonic Science. Low background scattering was ensured using a block collimation system; all the samples were taken from SPE films obtained by the SPE preparation. 2D Spectra were radially averaged using invoking self-developed plugins for ImageJ, corrected for both absorption and primary beam intensity, respectively. The q-range was normalized using based on crystalline silver behenate ($AgC_{22}H_{43}O_2$) as standard.

Atomic Force Microscopy (AFM)

For the measurement by means of atomic force microscopy (AFM), the polymer electrolytes were each spin-coated on a silicon wafer. For this, a solution of a block copolymer with the respective LiTFSI-content in acetonitrile (concentration = 3wt%; 0.04857 g of block copolymer/LiTFSI in 1.572 g (2 mL) acetonitrile) was prepared made and spin-coated using a K.L.M. Spin-Coater SCC-200. The rotation speed was accelerated from 0 to 100 rps (rounds per seconds) within 10 seconds and held constant for 90 seconds at 100 rps. The polymer solution was added dropwise (4*7 µL) at 100 rps within the first 30 seconds. Afterwards, the wafer was placed in an oven at 110 °C under reduced pressure ($1*10^{-3}$ mbar) for 6.5 days to assure a complete nanophase separation.

AFM measurements were then performed with a Cypher ES AFM system (Oxford Instruments, UK) using Tap150Al-G soft tapping tips (Budget Sensors, Bulgaria), where all the samples were measured under argon atmosphere and at a sample temperature of 32°C.

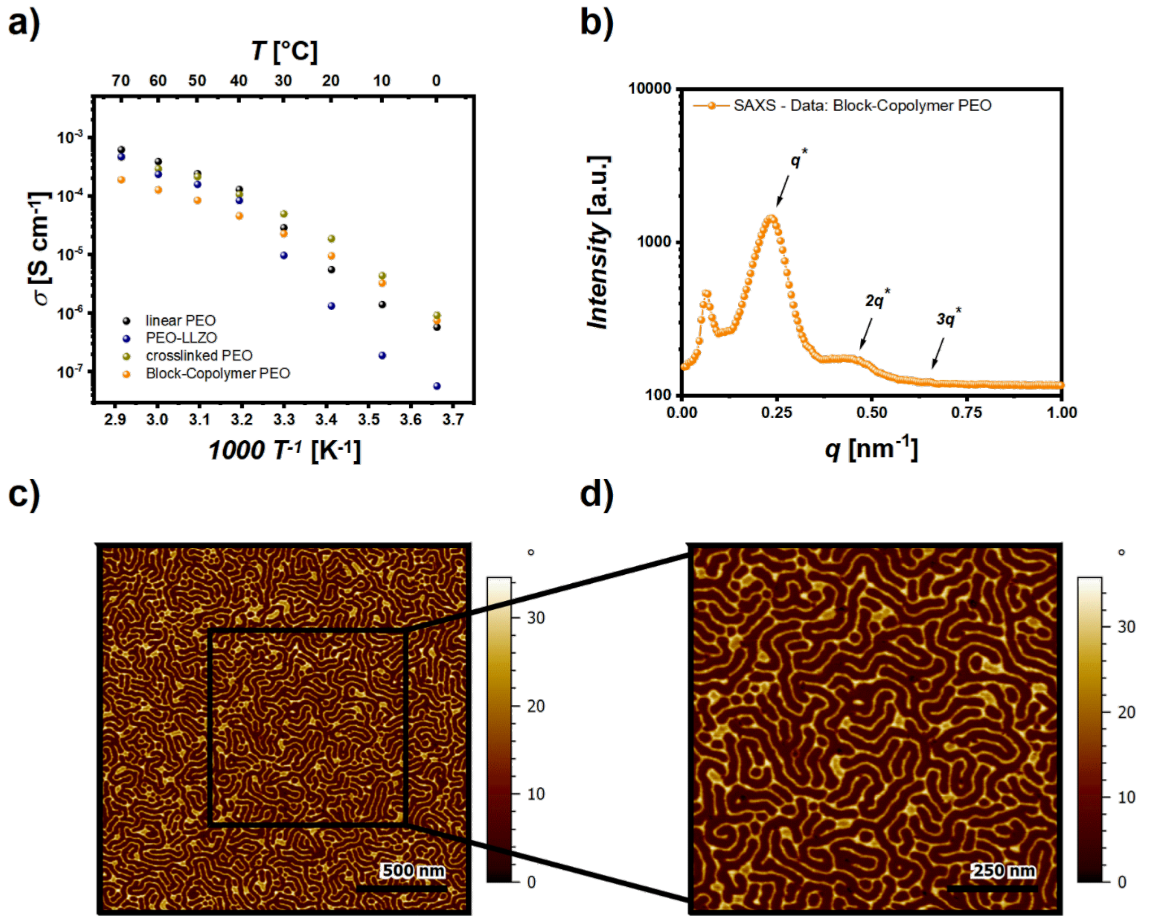

Figure S1: Transport properties of different PEO-based polymer electrolytes and morphology of the block copolymer a) ionic conductivity for all different polymers, b) SAXS data for the block copolymer c) and d) AFM phase images of distorted lamellae structure of the block copolymer.

Figure S1a) exhibits the measured ionic conductivities of the different selected SPEs. The un-cross-linked linear PEO has the highest conductivity at elevated temperature, however the ionic conductivity suffers from the presence of crystalline domains below ~40 °C. The PEO-LLZO hybrid electrolyte reveals similar behavior at higher temperature but displays an even increased ionic conductivity drop below ~40 °C. The crosslinking of the PEO-chains significantly reduces crystallinity resulting in inhibition of a conductivity drop and higher ionic conductivity at reduced

temperature. Though, at elevated temperature a slightly reduced ionic conductivity is observed. The formation of a nanostructured side-chain block copolymer was described in detail in earlier work.[7,8] The introduction of a robust second block increases the mechanical property of the polymer. However, the ionic conductivity is reduced in comparison to linear PEO. Note, that approximately 50% of the volume is isolating polystyrene, which is not participating in the ion transport. **Figure S1b)-d)** visualize the nanophase separation of the block copolymer by means of SAXS and AFM. The presence of more than one peak in SAXS data (**Figure S1b))** indicates structured morphology. The ratio of q at the peaks can be used to estimate the kind of structure and indicate a lamellae structure. By using **equation** (19), also the domain size can be estimated (~27 nm):

$$d = \frac{2\pi}{q^*} \tag{19}$$

Moreover, AFM phase images were recorded to visualize the nanostructured morphology and clearly corroborate the findings of the SAXS data. **Figure S1c)** and **d)** show the recorded AFM phase image, where **d)** shows a magnification of the inset in **Figure S1c)**. For the phase images, the sample is measured in tapping mode with a constant oscillation frequency applied. When the tip interacts with the sample surface, this oscillation frequency is shifted. Apart from additional influences from adhesion forces and long-range electrical forces, the major reason for this phase shift is the local elasticity of the sample. Hard surfaces show a low phase shift (dark in the phase image) while softer areas show an increased phase shift (lighter areas in the phase image).

Two phases can be readily distinguished from their phase (**Figure S1c)** and **d)**) and also from their topography contrast: The areas with a high phase shift (softer and/or more adhesive phase) appear to be slightly lower in the topography while the harder, less sticky areas which show a

lower phase shift are elevated. In total these differences are small (e.g. when comparing differences to polymer-particle mixtures, which have much higher differences in elasticity), but the two different phases can be distinguished unambiguously and verify a distorted lamellae structure in typical Turing pattern. Both phases appear as long strands with very consistent width. The width of the phases was determined from cross cuts of the phase image. The widths of each strand were determined at 90° to the orientation of the strand using the width at half maximum of the peak (width at half minimum for the softer phase), resulting in a width of the harder phase of roughly 18-25 nm and of about 10-15 nm for the softer phase. As these widths are near the resolution limit of the AFM tip, it is possible, that the size of the phases is slightly over- or underestimated. In summary, the measured widths are in a comparable range to the average domain spacing of 27 nm measured by SAXS, as the phases do not have an ordered structure.

Current sweep experiments with different sweep rates

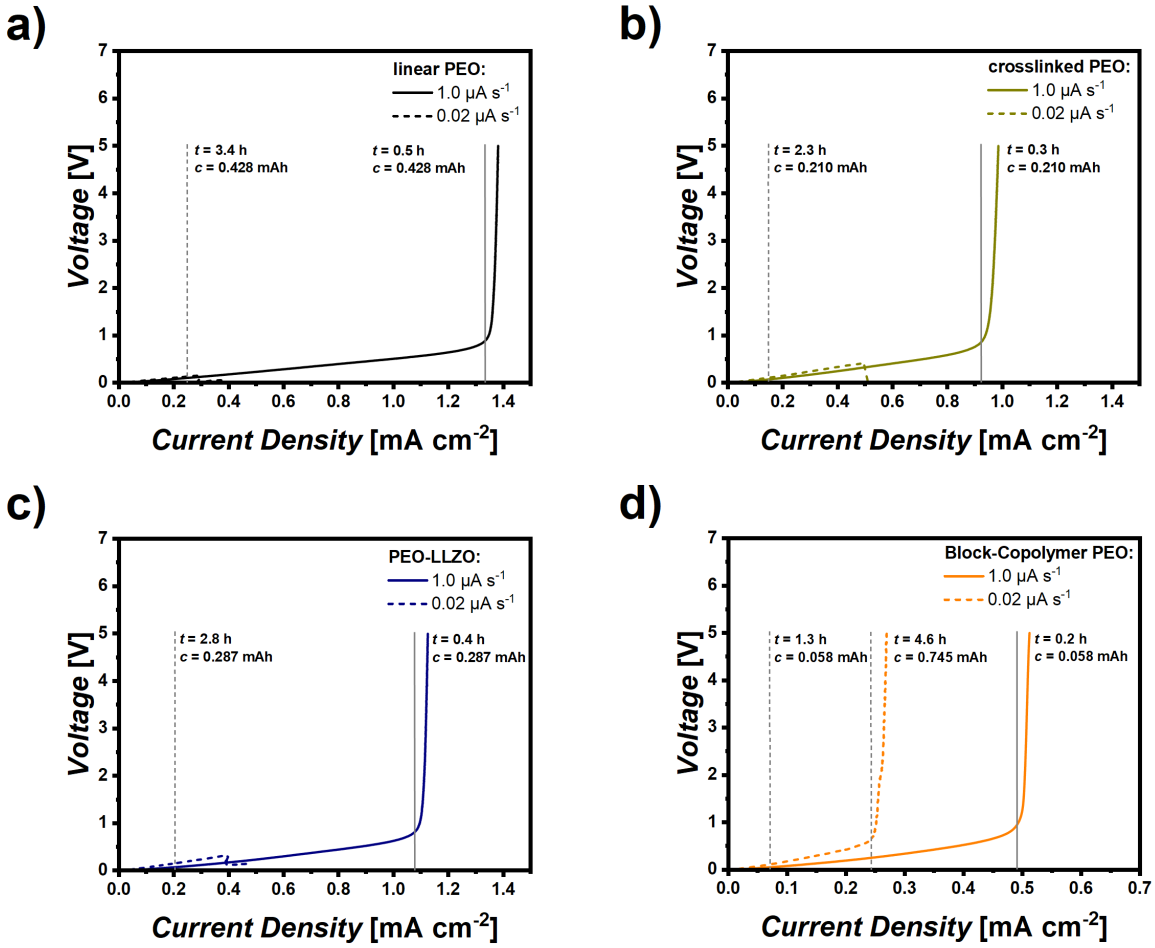

Figure S2: Determination of equivalent amount of plated lithium for current scan experiments with a fast and a slow scan rate in comparison for a) linear PEO, b) crosslinked PEO, c) PEO-LLZO hybrid electrolyte and d) block-copolymer electrolytes.

Figure S2 shows the current scan experiments for the four different electrolytes with a sweep rate of 1.0 µA s^{-1} (solid line) and 0.02 µA s^{-1} (dashed line). It is observed that only the block-copolymer PEO electrolyte displays a steep voltage increase for a current scan with a sweep rate of 0.02 µA s^{-1} reflecting a transport-limited LCD. For all the other electrolytes at a slow sweep rate of 0.02 µA s^{-1} sudden short circuits were observed resulting from electrolyte rupture due to lithium protrusions.

Moreover, the amount of plated lithium was calculated shortly before the LCD was determined at increased sweep rate (1.0 µA s^{-1}) and compared to the time as well as current density at which this amount of plated lithium was reached in the current scan with slow sweep rate (0.02 µA s^{-1}). **Table S3**, **Table S4**, **Table S5** and **Table S6** briefly summarize the calculated values for the different electrolytes. Only for the block-copolymer PEO a third value was calculated, which represents the amount of plated lithium shortly before the LCD is measured in the current scan with a slow sweep rate once more underlining the effect of the mechanical properties on the determination of the LCD.

Table S3: Overview about the amount of plated lithium for the PEO-based block-copolymer.

entry	solid polymer electrolyte	capacity [mAh]	time [h]
**1**	Block-Copolymer PEO (fast sweep rate)	**0.058**	0.2
**2**	Block-Copolymer PEO (slow sweep rate)	0.745	4.6
**3**	Block-Copolymer PEO (slow sweep rate)	**0.058**	1.3

Table S4: Overview about the amount of plated lithium for the linear PEO.

entry	solid polymer electrolyte	capacity [mAh]	time [h]
**1**	linear PEO (fast sweep rate)	**0.428**	0.5
**2**	linear PEO (slow sweep rate)	**0.428**	3.4

Table S5: Overview about the amount of plated lithium for the crosslinked PEO.

entry	solid polymer electrolyte	capacity [mAh]	time [h]
**1**	crosslinked PEO (fast sweep rate)	**0.210**	0.3
**2**	crosslinked PEO (slow sweep rate)	**0.210**	2.3

Table S6: Overview about the amount of plated lithium for the PEO-LLZO hybrid electrolyte.

entry	solid polymer electrolyte	capacity [mAh]	time [h]
**1**	PEO-LLZO (fast sweep rate)	**0.287**	0.4
**2**	PEO-LLZO (slow sweep rate)	**0.287**	2.8

Open circuit voltage (OCV) during conditioning-step

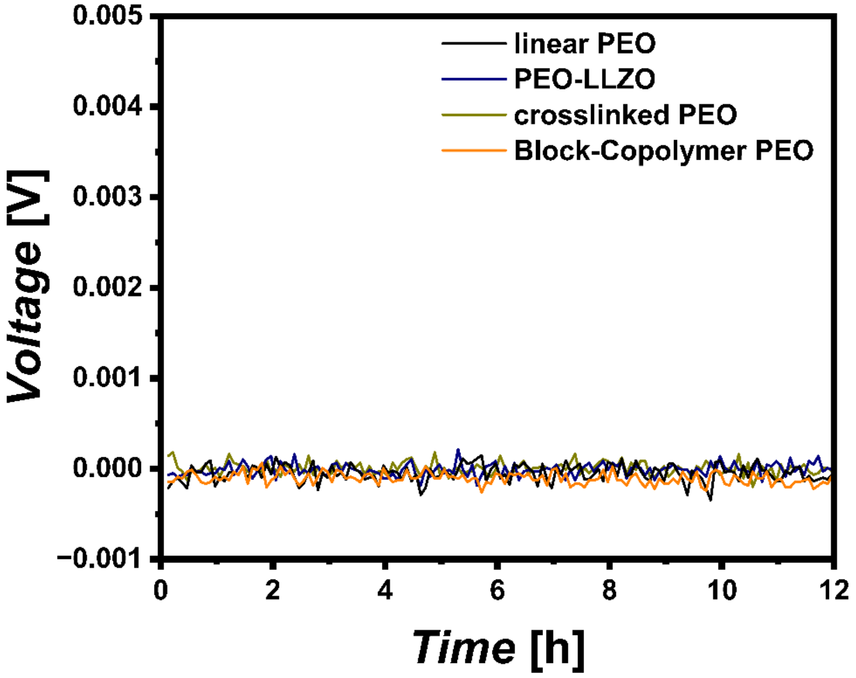

Figure S3: Open circuit voltage (OCV) recorded during the 12-hour conditioning-step before an experiment starts.

Electrochemical impedance spectroscopy

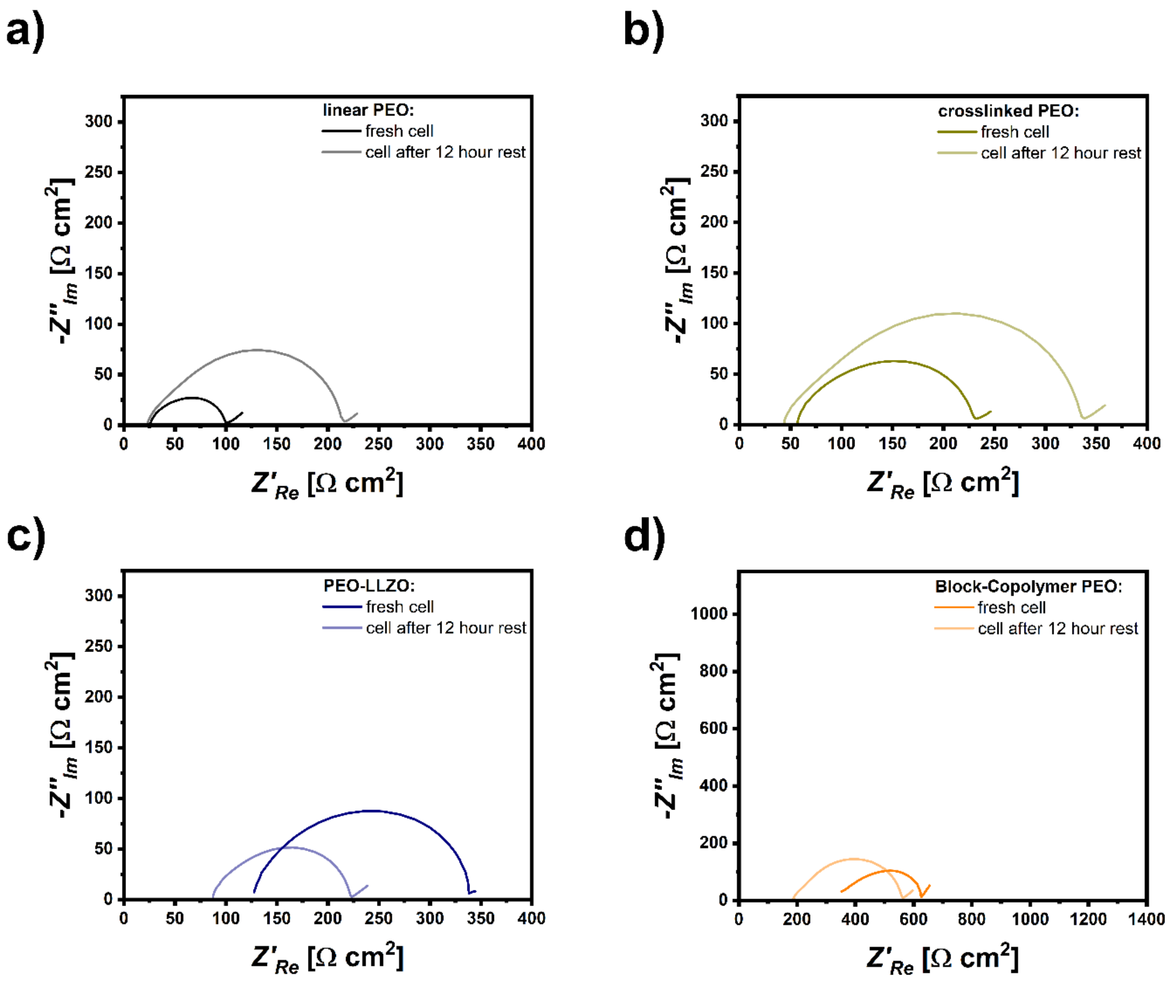

Figure S4: Impedance data before and after a 12-hour conditioning-step before conducting a current scan experiment with a sweep rate of 1.0 µA s^{-1}.

Figure S4 shows the impedance data of a fresh cell compared to the same cell after a 12-hour rest-step. It was assumed that an equilibrium was achieved after 12 hours. For all electrolytes a decrease in bulk electrolyte resistance (R_{bulk}, first x-intercept) ranging from 3 to 111 Ω cm^2 (**Table S7**, depending on the SPE and technical inconsistencies) can be observed after the 12-hour rest-step resulting from temperature equilibration since the fresh cells were in a rather undefined temperature state and the bulk electrolyte resistance (R_{bulk}) is highly temperature-dependent.

Moreover, the impedance data reflect that the overall resistance is increasing for all electrolytes except the PEO-LLZO hybrid electrolyte (**Figure S4c), Table S7**). This increase in overall resistance is caused by the formation of interfaces and interphases such as the solid electrolyte interface (SEI). For the PEO-LLZO hybrid electrolyte (**Figure S4c)**) the overall resistance is decreased from 211 Ω cm^2 to 135 Ω cm^2 which might be due to the chemical nature of the hybrid electrolyte and the incorporated particles which could potentially be beneficial for interphase formation and charge transfer processes.[9] These effects should be investigated in more detail in material-focused studies, but would be beyond the scope of this study.

Table S7: Bulk electrolyte resistance (R_{bulk}) as well as the total resistance (R_{total}) of the cells before and after the 12-hour conditioning-step.

	R_{bulk} (before rest) [Ω cm^2]	R_{bulk} (after rest) [Ω cm^2]	R_{total} (before rest) [Ω cm^2]	R_{total} (after rest) [Ω cm^2]
**linear PEO**	26	23	83	195
**crosslinked PEO**	56	43	177	295
**PEO-LLZO**	127	88	211	135
**Copolymer PEO**	331	185	297	375

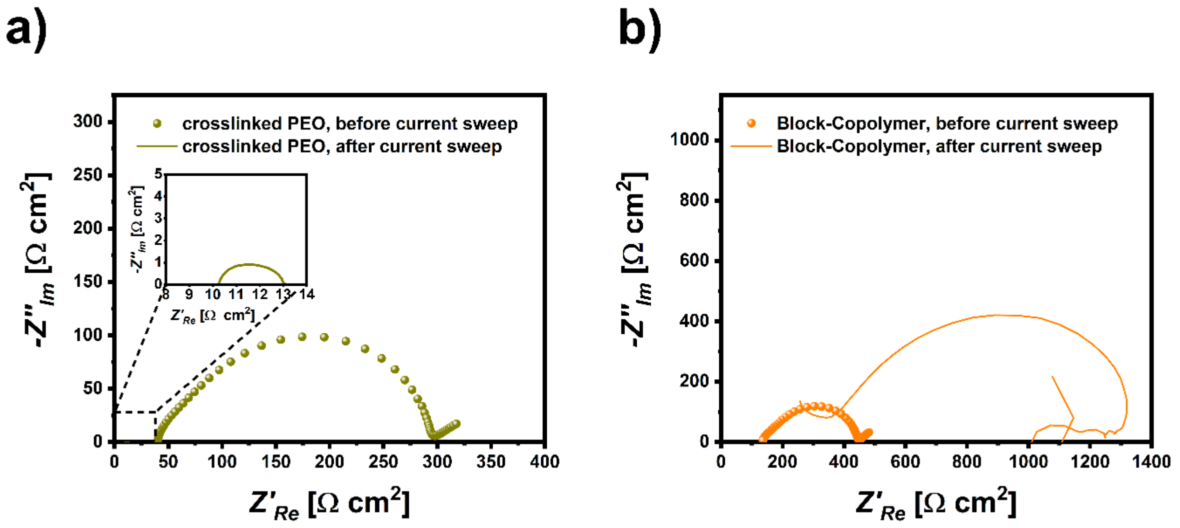

Figure S5: Impedance spectra of Li||Li cells before and after a current scan at a sweep rate of 0.02 µA s^{-1}, using a) crosslinked PEO and b) block copolymer as solid polymer electrolyte.

Table S8: Total resistance (R_{total}) of Li||Li cells before and after a current scan at a sweep rate of 0.02 µA s^{-1} reflecting the different reasons for failure during LCD determination.

	$\boldsymbol{R_{total}}$ **(before current sweep) [Ω cm²]**	$\boldsymbol{R_{total}}$ **(after current sweep) [Ω cm²]**
**crosslinked PEO**	257	3
**Block-Copolymer PEO**	315	~1028

References

(1) Newman, J. S.; Balsara, N. P. *Electrochemical systems*, 4th ed.; The ECS Series of Texts and Monographs; Wiley: Hoboken, NJ, 2021.

(2) Climent, V.; Feliu, J. M. Cyclic Voltammetry. In *Encyclopedia of interfacial chemistry*: *Surface science and electrochemistry*; Elsevier Science, 2018; pp 48. DOI: 10.1016/B978-0-12-409547-2.10764-4.

(3) Bielefeld, A.; Weber, D. A.; Rueß, R.; Glavas, V.; Janek, J. Influence of Lithium Ion Kinetics, Particle Morphology and Voids on the Electrochemical Performance of Composite Cathodes for All-Solid-State Batteries. *J. Electrochem. Soc.* **2022**, *169* (2), 20539. DOI: 10.1149/1945-7111/ac50df.

(4) Xu, W.; Wang, J.; Ding, F.; Chen, X.; Nasybulin, E.; Zhang, Y.; Zhang, J.-G. Lithium Metal Anodes for Rechargeable Batteries. *Energy Environ. Sci.* **2014**, *7* (2), 513. DOI: 10.1039/C3EE40795K.

(5) Kuchel, P. W.; Pagès, G.; Nagashima, K.; Velan, S.; Vijayaragavan, V.; Nagarajan, V.; Chuang, K. H. Stejskal–tanner equation derived in full. *Concepts Magn. Reson. Part A* **2012**, *40A* (5), 205. DOI: 10.1002/cmr.a.21241.

(6) Sinnaeve, D. The Stejskal–Tanner equation generalized for any gradient shape—an overview of most pulse sequences measuring free diffusion. *Concepts Magn. Reson. Part A* **2012**, *40A* (2), 39. DOI: 10.1002/cmr.a.21223.

(7) Butzelaar, A. J.; Röring, P.; Hoffmann, M.; Atik, J.; Paillard, E.; Wilhelm, M.; Winter, M.; Brunklaus, G.; Theato, P. Advanced Block Copolymer Design for Polymer Electrolytes: Prospects of Microphase Separation. *Macromolecules* **2021**, *54* (23), 11101. DOI: 10.1021/acs.macromol.1c02147.

(8) Butzelaar, A. J.; Röring, P.; Mach, T. P.; Hoffmann, M.; Jeschull, F.; Wilhelm, M.; Winter, M.; Brunklaus, G.; Théato, P. Styrene-Based Poly(ethylene oxide) Side-Chain Block Copolymers as Solid Polymer Electrolytes for High-Voltage Lithium-Metal Batteries. *ACS Appl. Mater. Interfaces* **2021**, *13* (33), 39257. DOI: 10.1021/acsami.1c08841.

(9) Kato, M.; Hiraoka, K.; Seki, S. Investigation of the Ionic Conduction Mechanism of Polyether/$Li_7La_3Zr_2O_{12}$ Composite Solid Electrolytes by Electrochemical Impedance Spectroscopy. *J. Electrochem. Soc.* **2020**, *167* (7), 70559. DOI: 10.1149/1945-7111/ab8478.
